# Expressed protein profile of a Tectomicrobium and other microbial symbionts in the marine sponge *Aplysina aerophoba* as evidenced by metaproteomics

**DOI:** 10.1038/s41598-018-30134-0

**Published:** 2018-08-07

**Authors:** Maryam Chaib De Mares, Diego Javier Jiménez, Giorgia Palladino, Johanna Gutleben, Laura A. Lebrun, Emilie E. L. Muller, Paul Wilmes, Detmer Sipkema, Jan Dirk van Elsas

**Affiliations:** 1 Microbial Ecology Cluster, Groningen Institute for Evolutionary Life Sciences (GELIFES), University of Groningen, Nijenborgh 7, 9747 AG Groningen, The Netherlands; 2 Department of Biological Sciences, Universidad de los Andes, Bogotá, Colombia; 3 Laboratory of Microbiology, Wageningen University, Wageningen, The Netherlands; 4 Luxembourg Centre for Systems Biomedicine, University of Luxembourg, Esch-sur-Alzette, Luxembourg; 5 Department of Microbiology, Genomics and the Environment, UMR 7156 UNISTRA – CNRS, Université de Strasbourg, Strasbourg, France

## Abstract

*Aplysina aerophoba* is an emerging model marine sponge, with a well-characterized microbial community in terms of diversity and structure. However, little is known about the expressed functional capabilities of its associated microbes. Here, we present the first metaproteomics-based study of the microbiome of *A*. *aerophoba*. We found that transport and degradation of halogenated and chloroaromatic compounds are common active processes in the sponge microbiomes. Our data further reveal that the highest number of proteins were affiliated to a sponge-associated Tectomicrobium, presumably from the family Entotheonellaceae, as well as to the well-known symbiont “*Candidatus* Synechococcus spongiarium”, suggesting a high metabolic activity of these two microorganisms *in situ*. Evidence for nitric oxide (NO) conversion to nitrous oxide was consistently observed for Tectomicrobia across replicates, by production of the NorQ protein. Moreover, we found a potential energy-yielding pathway through CO oxidation by putative Chloroflexi bacteria. Finally, we observed expression of enzymes that may be involved in the transformation of chitin, glycoproteins, glycolipids and glucans into smaller molecules, consistent with glycosyl hydrolases predicted from analyses of the genomes of Poribacteria sponge symbionts. Thus, this study provides crucial links between expressed proteins and specific members of the *A*. *aerophoba* microbiome.

## Introduction

Sponges (phylum Porifera) have been documented as complex environments hosting microbial representatives from all three domains of life^1,2^. Especially, sponges with poor irrigation systems contain large numbers of bacteria and are known as high-microbial abundance (HMA) sponges or “bacteriosponges”, as opposed to well-irrigated sponges that are known as low-microbial abundance (LMA) sponges^3^. Therefore, in particular in HMA sponges, many of the associated microbes are understood as an essential part of the sponge. This typically includes bacterial and archaeal clades found only in marine sponges. These clades are taken to represent “sponge-specific clusters”, referring to groups of at least three sequences that: (i) were recovered from different sponge species and/or different geographic locations, (ii) are more closely related to each other than to any other sequence from non-sponge sources, and (iii) cluster together independently of the method used^4,5^.

Marine sponges from the genus *Aplysina* are classified as “bacteriosponges”, which host bacterial cells in densities between 10^8^–10^10^ per gram of sponge wet weight, resulting in many cases in up to 40% of the sponge biomass being composed of bacterial cells^6^. In particular *Aplysina aerophoba* is an emerging model sponge species^7^; its bacterial diversity was found to include at least 36 different phyla. Of these, Bacteroidetes, Cyanobacteria, Proteobacteria, Actinobacteria and representatives of the poorly characterized phyla Acidobacteria, Chloroflexi, Planctomycetes and Verrucomicrobia, as well as the candidate phylum Poribacteria^8^, are often abundant in the sponge microbiomes^2^.

Even though the prokaryotic diversity and composition within *A*. *aerophoba* is known at a rather broad level, we understand much less regarding the active functional profile of the sponge-associated microbiome. This lack of information is not restricted to *A*. *aerophoba*, but extends to sponges in general. The reasons are twofold: most sponge-associated microbes have not been brought into culture, and culture-independent ‘omics’ methods have only recently started to be applied to sponges. The latter methods can provide us with either an overview of functions present (*i*.*e*. through metagenomics or single cell genomics); or of realized (active) functions (using for instance metatranscriptomics and/or metaproteomics)^9^. Pioneering single-cell genomics work on the *A*. *aerophoba* microbiome revealed the genetic content of members of the symbiotic candidate phylum Poribacteria^10,11^. Gene arrays based on DNA hybridization have shown the genetic potential for bacterial ammonia oxidation (*amoA*), ammonification (*ureC* subunit of urease), and archaeal autotrophic carbon fixation (via propionyl-CoA-carboxylase, *pcc*) in *A*. *aerophoba* and a number of other HMA and LMA sponges^12^. A study based on a metagenomics approach was recently conducted for *A*. *aerophoba*^13^ where defense-related functions such as CRISPR-Cas systems were explored. In addition, a draft genome of the sponge-associated “*Candidatus* Synechococcus spongiarum” from *A*. *aerophoba*^14^ is now available.

The only study in which metaproteomics was applied to sponges thus far investigated the interactions between the LMA sponge *Cymbastela concentrica* and its microbial community^15^. Nitrogen metabolism of bacterial and archaeal symbionts was found to be closely linked to the metabolism of the sponge host, which secretes ammonium. Specifically, the authors found expression of the ammonia monooxygenase proteins (AmoB and C) and an ammonia transporter (AmtB) in the *C*. *concentrica* microbiome. The occurrence of key microbiome metabolic activities, such as (anaerobic) sulfur and nitrogen transformations (as energy-generating pathways) in sponges has been controversial^16^. Preliminary evidence has pointed to sulfur reduction^17,18^ and denitrification^15^.

In order to provide testable hypotheses about the active metabolic functions of the microbial communities that are associated with *A*. *aerophoba*, we performed a metaproteomics-based analysis on the basis of freshly sampled sponge material. Our results show the expression of transporters for host-derived nutrients and halogenated aromatic compounds. In addition, our data allow us to suggest that the assimilation of carbon is common in both aerobic and anaerobic conditions within the sponge environment. Moreover, we could confirm the presence and high metabolic activity of a Tectomicrobium in the sponge, in addition to the well-known symbiotic Cyanobacteria. These observations are highly relevant for our understanding of the metabolic activity of key symbionts and their functions within the *A*. *aerophoba* microbiome.

## Results

### Overview of the *Aplysina aerophoba* microbial metaproteome

**Figure 1:**
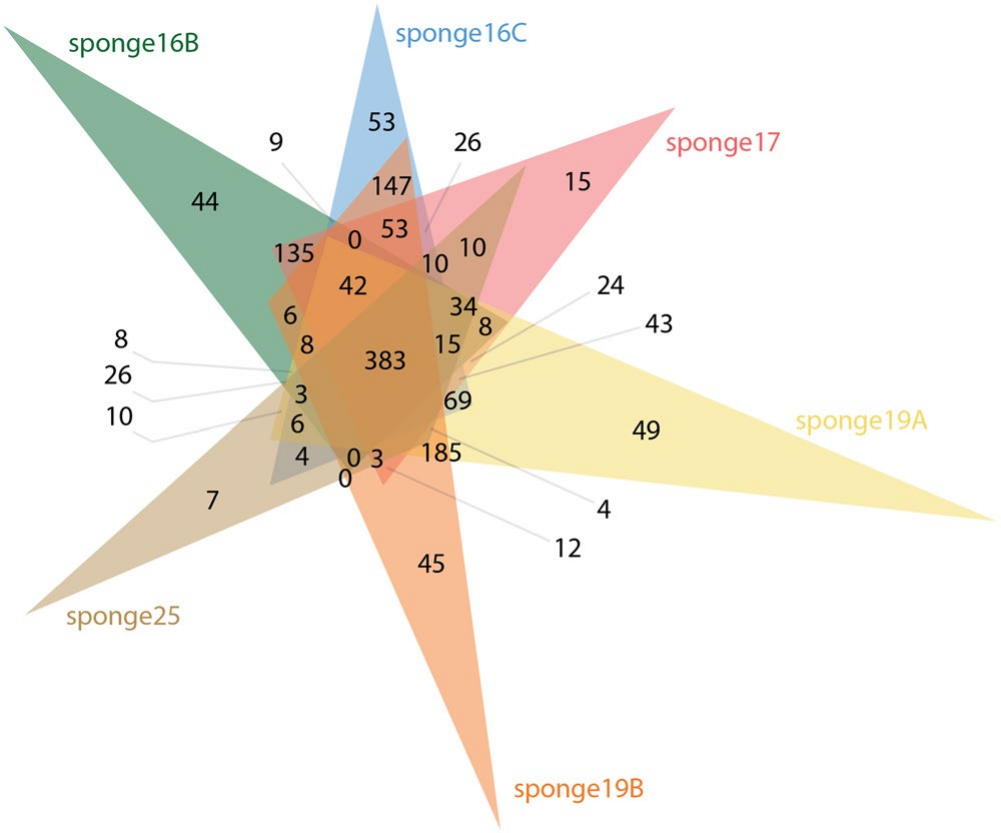
Venn diagram depicting number of proteins shared and unique to each of the *Aplysina aerophoba* sponge replicates used in this study.

Four individuals of *Aplysina aerophoba* (16, 17, 19, 25) and four samples of surrounding seawater (5, 6, 7, 8) were collected in the Mediterranean Sea (see Methods). Sponge individuals 16 and 19 were processed in technical replicates (Table 1). *De novo* peptide sequencing generated between 1,609 and 4,560 peptide sequences for each of the sponge samples. A total of 2,536 peptide sequences were obtained in the pooled seawater samples (Table 1). The resulting peptides were matched against predicted proteins from two metagenomic assemblies, one per habitat. The number of peptides per sponge sample with a match in the database with predicted proteins in assembled metagenomes from *A*. *aerophoba* (782,998 predicted proteins) and surrounding seawater (370,455 predicted proteins) was in a range of 72.3% to 76.7% of the sequenced peptides. In contrast, the number of peptides with a match in the database for seawater samples was rather low, between 9.9 and 27.9%. From this initial set of expressed proteins, we could match between 245 and 757 protein sequences from each of the sponge samples to the corresponding metagenomics datasets (Table 1), adding up to a total of 3,226 proteins. Of these, 383 proteins were shared among all individual sponge samples, and up to 185 proteins were shared between any two samples from the same individual (Fig. 1). In the seawater, a total of 222 common proteins was identified in the four samples.

**Table 1:** Overview of sequenced peptides and proteins traceable to the metagenomics datasets per sample and habitat.

Habitat	Sponge (*Aplysina aerophoba*)						Sea water			
Sample ID	16B	16C	17	19A	19B	25	sw5	sw6	sw7	sw8
Number of *de novo* sequenced peptides	2579	3639	3342	4560	3944	1609	1035	629	509	363
Number of peptides with a match in the database	1864	2778	2458	3497	2969	1167	289	107	116	36
Percentage of peptides with a match in the database (%)	72.3	76.3	73.5	76.7	75.3	72.5	27.9	17.0	22.8	9.9
Number of identified proteins in database	390	641	523	757	670	245	108	45	52	17
Number of identified proteins with >40% peptide coverage	85	63	64	95	75	37	0	0	4	0
Functionally annotated proteins* (%)	166 (42.6)	329 (51.3)	234 (44.7)	422 (55.7)	391 (58.4)	131 (53.5)	76 (70.4)	27 (60)	32 (61.5)	8 (47.1)

*Numbers of functional characterizations based on assignments using BlastKOALA^65^.

**Figure 2:**
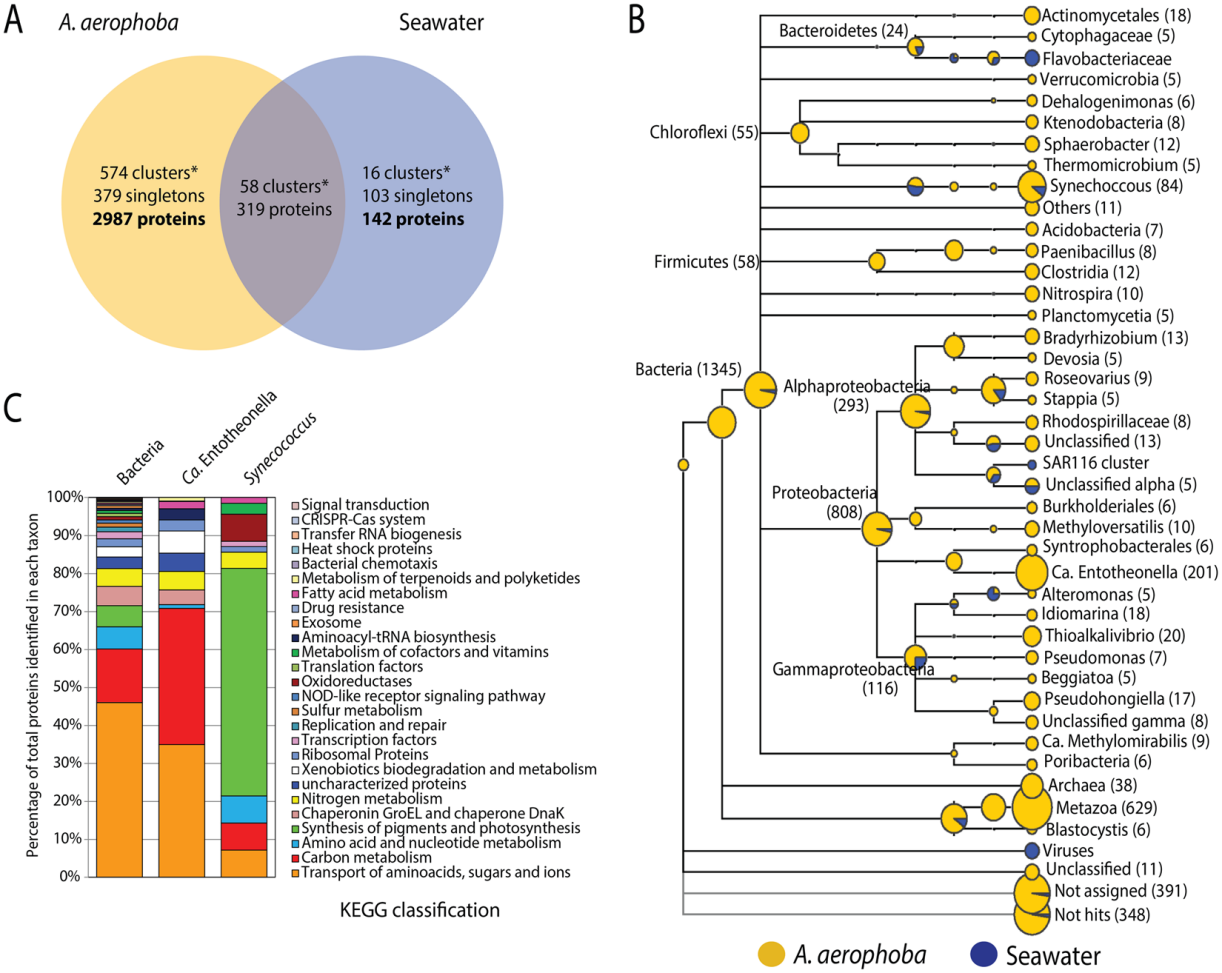
Taxonomic and functional annotations of proteins in the sponge *A*. *aerophoba* and surrounding seawater. (**A**) Venn diagram showing the number of orthologous clusters (*), singletons and total unique proteins per habitat. (**B**) Taxonomic assignment of 2987 (unique in the sponge *A*. *aerophoba*, yellow) and 142 (unique in sea water, blue) proteins by the LCA algorithm. Numbers in brackets correspond to proteins affiliated to each taxon. (**C**) Functional assignment using KEGG orthology (KO) of proteins affiliated to the Bacteria domain, and the genera contributing the largest number of unique proteins in the sponge *A*. *aerophoba*.

**Figure 3:**
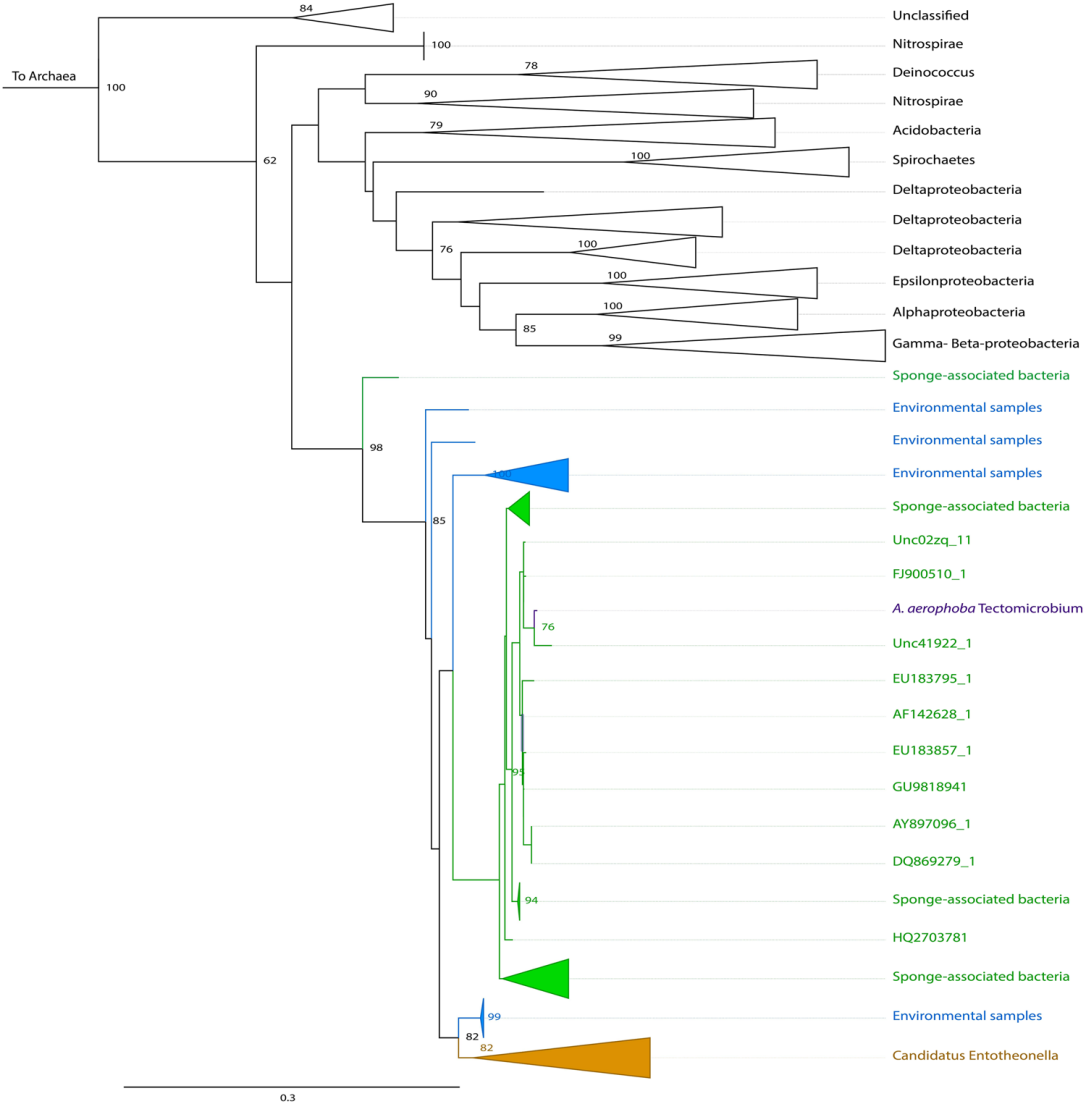
Maximum likelihood phylogeny of the 16S rRNA marker gene depicting the phylogenetic position of the sequence of interest (*A*. *aerophoba* Tectomicrobium) within the candidate phylum Tectomicrobia^20^. The green cluster includes sponge-associated Tectomicrobia sequences. Sequences derived from other environmental samples and which are part of the phylum Tectomicrobia are shown in blue. The orange cluster contains sequences affiliated to the *Entotheonella* genus.

**Figure 4:**
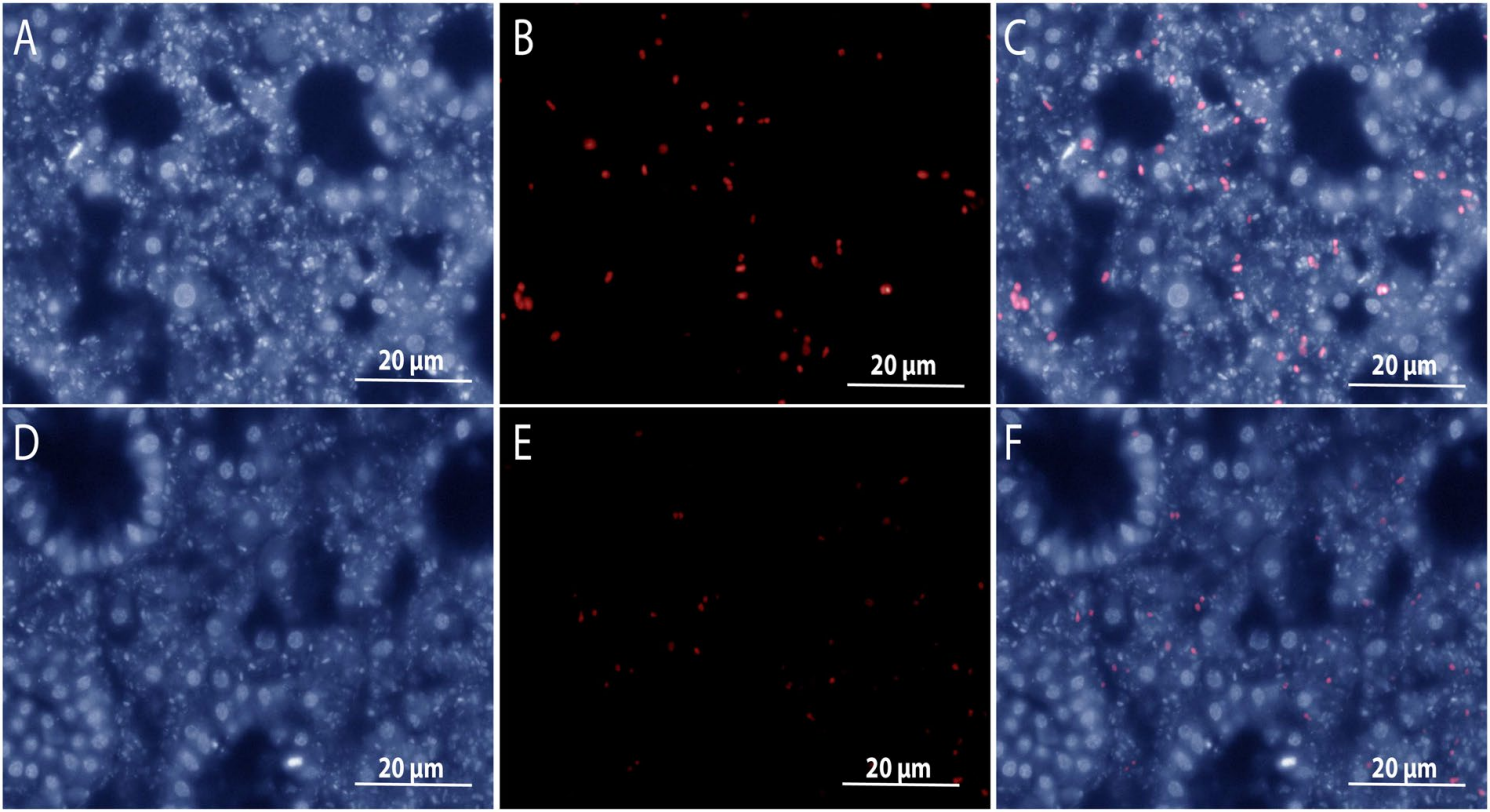
CARD-FISH detection of Tectomicrobia bacteria in tissue sections of the sponge *A*. *aerophoba*. (**A**,**D**) DAPI-stained cells. (**B**,**E**) Corresponding tissue sections hybridized with Tectomicrobia Probe 1 (**B**) and Probe 2 (**E**) visualized with deep red filter (exposure time 100 ms). (**C**,**F**) Overlay of DAPI-staining and the fluorescent image. All probe-labelled cells correspond to a DAPI signal. (Scale bars: 20 μm).

In order to explore the differences on the expressed protein profiles between the sponge and seawater, we performed a clustering analysis^19^ using the detected and pooled proteins in each habitat. We found 574 orthologous clusters and 379 singletons unique to the sponge, in contrast to 16 clusters and 103 singletons in the seawater (Fig. 2A). A total of 2,987 proteins was uniquely encountered in the sponge-derived proteomes, whereas 142 were unique to the seawater. The subset of proteins unique to each habitat (sponge vs seawater) was affiliated to the lowest reliable taxonomic category using the Low Common Ancestor (LCA) algorithm (Fig. 2B). Proteins assigned to Metazoa (629), to Archaea (38), not assigned by the LCA algorithm (391), or with no BLASTP hits (348) were not analyzed further. The 1,345 proteins that were taxonomically identified as bacterial were subsequently analyzed in regard to their predicted function. The phyla contributing the largest number of proteins in the sponge were Proteobacteria (808), Cyanobacteria (84), Firmicutes (58), Chloroflexi (55) and Bacteroidetes (24). The classes Alphaproteobacteria (293) and Gammaproteobacteria (116) were the largest contributors within the Proteobacteria phylum. Proteins assigned to the candidate phyla Tectomicrobia (201), Poribacteria (6) and the candidate division NC10 (9) were also found exclusively within the sponge protein repertoire. Consistent with this pattern, we found the largest number of spectral counts for bacterial phyla Acidobacteria, Actinobacteria, Bacteroidetes, Chloroflexi, Cyanobacteria, Firmicutes and Proteobacteria (classes Alpha and Gamma) (see Supplementary Table S1). At a lower taxonomic level, the genera contributing the largest number of unique proteins in the sponge were *Entotheonella* (201) (candidate phylum Tectomicrobia), *Synechococcus* (84) (Cyanobacteria), *Thioalkalivibrio* (20), *Idiomarina* (18) and *Pseudohongiella* (17) (Gammaproteobacteria). The remaining identified genera contributed <15 proteins each. Complementary analysis based on reads matching the 16S rRNA gene extracted from the sequenced metagenomes (see Supplementary Fig. S1) and gene markers from the assembled contigs (see Supplementary Fig. S2) showed that the vast majority of organisms included in our metagenomics database were of bacterial origin (91%). Some of the major groups found, coincided with the taxa detected using the LCA algorithm, namely Proteobacteria (36%), with a dominance of Alpha-(10%) and Gammaproteobacteria (14%), and Chloroflexi (17%). The Deltaproteobacteria and unclassified bacteria made up to 4% and 10% of the contigs of the database, respectively. Although the genus *Entotheonella* was not explicitly found with the gene marker analysis performed with Phylosift, two partial Entotheonellaceae 16S rRNA gene sequences were recovered from our metagenomic assembly (see Supplementary Table S2). Depending on the database used, Entotheonellaceae sequences are classified within the Deltaproteobacteria (Green Genes), as it was originally described^20^, or within the Tectomicrobia (SILVA), as it is currently accepted (see Supplementary Table S2). These two fragments overlapped with 45 bp and constitute a full-length 16S rRNA marker gene, which had a relative abundance of 0.4 ± 0.01% in the sponge metagenomes. Phylogenetic analyses of the 16S rRNA marker gene revealed the position of this sequence within sponge-associated Tectomicrobia, not within the genus *Entotheonella sensu stricto* (Fig. 3). The *A*. *aerophoba*-derived Entotheonellaceae consensus sequence is clustered within the sponge-associated Tectomicrobia reported previously^20^. Hybridization with probes designed for CARD-FISH revealed the presence of the sponge-associated Tectomicrobia scattered throughout the *A*. *aerophoba* tissue, both with probe-1 (Fig. 4A–C) and probe-2 (Fig. 4D–F).

### Functional annotation of proteins in *Aplysina aerophoba* microbiome based on KO identifiers

Roughly half of the expressed proteins had no KEGG orthology (KO) assignment (Table 1), suggesting the presence of many unrecognized functions in the microbial communities of the two habitats. We thus focused on the subset of bacterial proteins found specifically in the sponge for the KO analysis. From the resulting 1,345 bacterial proteins, 806 (60%) could be assigned to a KO category (Fig. 2C). The entire list of bacterial proteins can be found as Supplementary Table S3. Considering the total amount of individual KO categories among these bacterial proteins present in multiple samples, 72% of these are validated in a minimum of three biological replicates. The KO group with the highest number of proteins was transport of amino acids, sugars and ions (371), followed by carbon metabolism (114), amino acid and nucleotide metabolism (47), photosynthesis (45), nitrogen metabolism (38) and degradation and metabolism of xenobiotics (22). Within the latter category, the expressed proteins were related to the conversion of carbon monoxide (CoxL and CoxM) and the degradation of aromatic compounds (PcaC, PcaD and carboxymethylenebutenolidase [EC: 3.1.1.45]). Eight proteins were found to be related to sulfur metabolism and one to a CasA protein within a CRISPR system.

A close examination of the top-20 KO identifiers in Bacteria based on absolute abundance, in a range of ten to 140 proteins, showed that the transport of peptides/nickel (K02035), sugars (K02027), amino acids (K01999), polyamines (e.g. putrescine and spermidine) (K02055), sulfonate, nitrate and taurine (K02051) were highly represented in the *A*. *aerophoba* bacterial metaproteome. Sixteen out of these 20 functional categories were present in four out of five individual samples. Moreover, we observed a high number of proteins affiliated with the chaperonin GroEL (K04077), next to others potentially involved in carbon (e.g. isocitrate and tricarballylate dehydrogenases; gluconolactonase) and nitrogen metabolism (e.g. formamidase and glutamine synthetase) (Table 2).

**Table 2:** Top 20 KEGG orthology protein functions affiliated to Bacteria in the sponge *A*. *aerophoba*.

KO	Function	Total Proteins	Replicates					
			16B	16C	17	19A	19B	25
K02035	ABC.PE.S; peptide/nickel transport system substrate-binding protein	140	3	25	11	56	39	6
K01999	LivK; branched-chain amino acid transport system substrate-binding protein	82	3	22	10	25	20	2
K04077	GroEL; chaperonin GroEL	58	14	4	12	8	9	11
K09969	AapJ; general L-amino acid transport system substrate-binding protein	37	2	7	6	6	11	5
K02055	ABC.SP.S; putative spermidine/putrescine transport system substrate-binding protein	37	0	8	3	13	12	1
K02051	ABC.SN.S; NitT/TauT family transport system substrate-binding protein	34	1	8	6	8	9	2
K02027	ABC.MS.S; multiple sugar transport system substrate-binding protein	25	2	4	1	10	7	1
K07080	uncharacterized protein	22	0	7	2	5	8	0
K02030	ABC.PA.S; polar amino acid transport system substrate-binding protein	20	0	6	0	7	7	0
K01455	Formamidase [EC:3.5.1.49]	17	0	6	1	6	4	0
K01053	Gluconolactonase [EC:3.1.1.17]	15	0	5	2	5	2	1
K00114	ExaA; alcohol dehydrogenase (cytochrome c) [EC:1.1.2.8]	14	0	3	2	3	3	3
K03530	HupB; DNA-binding protein HU-beta	14	11	0	1	0	0	2
K03520	CoxL; carbon-monoxide dehydrogenase large subunit [EC:1.2.7.4]	14	11	0	1	0	0	2
K00031	Idh1; isocitrate dehydrogenase [EC:1.1.1.42]	13	1	1	1	2	8	0
K05377	CpeB; phycoerythrin beta chain	12	2	2	2	2	2	2
K01011	TST; thiosulfate/3-mercaptopyruvate sulfurtransferase [EC:2.8.1.1 2.8.1.2]	12	0	8	1	0	3	0
K05376	CpeA; phycoerythrin alpha chain	11	2	2	2	2	1	2
K00024	Mdh; malate dehydrogenase [EC:1.1.1.37]	10	2	1	0	4	1	2
K03519	CoxM; carbon-monoxide dehydrogenase medium subunit [EC:1.2.7.4]	10	1	6	0	1	2	0

Numbers indicate abundance.

A KO classification conducted for the two genera to which most proteins were assigned in the sponge (a “*Candidatus* Entotheonella sp.” related bacterium (Fig. 2C and Table 3) and “*Candidatus* Synecochoccus spongiarum”; Fig. 2C) revealed a number of potential key activities. Thus, the former organism was found to express transporters for peptide/nickel (14 proteins), sugars (6), amino acids (5) and urea (1). In addition, four nitric oxide reductases (NorQ) (Table 3) were found. We observed that in 58% of the cases, KO categories assigned to proteins of an Entotheonellaceae bacterium were present in at least three out of five replicates. In the case of *Synecocchocus*, the majority of proteins was related to the synthesis of pigments for photosynthesis, as well as to oxidation/reduction reactions (oxidoreductases). We also examined the proteins that were taxonomically assigned to a bacterium similar to “*Candidatus* Methylomirabilis oxyfera” and Poribacteria. All nine proteins of the “*Candidatus* Methylomirabilis oxyfera”-like bacterium matched putative aldehyde dehydrogenases at between 64 and 68% amino acid identity. Similarly, the six proteins matching Poribacteria have putative oxidoreductase activities (identity >90%). The entire lists of proteins assigned to Entotheonellaceae and “*Candidatus* Synecochoccus spongiarum”, and “*Candidatus* Methylomirabilis oxyfera” and Poribacteria can be found online as Supplementary Table S4 and Supplementary Table S5, respectively.

**Table 3:** Functional assignment (KEGG orthology) of proteins affiliated to a putative Tectomicrobium in the sponge *A*. *aerophoba*.

KO	Function	Total Proteins	Replicates					
			16B	16C	17	19A	19B	25
K02035	ABC.PE.S; peptide/nickel transport system substrate-binding protein	14	1	1	8	2	2	0
K13796	CobZ; tricarballylate dehydrogenase	9	0	1	4	3	1	0
K01053	Gluconolactonase [EC:3.1.1.17]	8	0	4	1	1	1	1
K00114	ExaA; alcohol dehydrogenase (cytochrome c) [EC:1.1.2.8]	8	0	2	1	2	2	1
K01652	Acetolactate synthase I/II/III large subunit [EC:2.2.1.6]	7	0	2	0	2	3	0
K01999	LivK; branched-chain amino acid transport system substrate-binding protein	7	0	3	0	3	1	0
K02027	ABC.MS.S; multiple sugar transport system substrate-binding protein	6	0	1	0	3	2	0
K09969	AapJ; general L-amino acid transport system substrate-binding protein	5	0	1	1	1	1	1
K07045	Uncharacterized protein	5	0	1	1	1	1	1
K00031	Idh1; isocitrate dehydrogenase [EC:1.1.1.42]	4	1	0	1	1	1	0
K04077	Chaperonin GroEL	4	1	0	2	1	0	0
K04748	Nitric oxide reductase NorQ protein	4	0	1	1	1	1	0
K02433	GatA; glutamyl-tRNA(Gln) amidotransferase subunit A [EC:6.3.5.6]	3	0	0	0	3	0	0
K02945	RP-S1; small subunit ribosomal protein S1	3	0	1	1	0	1	0
K01061	Carboxymethylenebutenolidase [EC:3.1.1.45]	3	0	0	1	0	1	1
K01607	PcaC; 4-carboxymuconolactone decarboxylase [EC:4.1.1.44]	3	3	0	0	0	0	0
K00059	FabG; 3-oxoacyl-[acyl-carrier protein] reductase [EC:1.1.1.100]	2	0	0	0	0	2	0
K02031	ABC.PE.A; peptide/nickel transport system ATP-binding protein	2	0	1	0	0	1	0
K00821	ArgD; acetylornithine/N-succinyldiaminopimelate aminotransferase [EC:2.6.1.11]	1	0	1	0	0	0	0
K00008	SorD; L-iditol 2-dehydrogenase [EC:1.1.1.14]	1	0	1	0	0	0	0
K14733	LimB; limonene 1,2-monooxygenase [EC:1.14.13.107]	1	0	1	0	0	0	0
K01455	Formamidase [EC:3.5.1.49]	1	0	1	0	0	0	0
K10823	OppF; oligopeptide transport system ATP-binding protein	1	0	1	0	0	0	0
K11959	UrtA; urea transport system substrate-binding protein	1	0	0	0	1	0	0

### ‘Abundant’ functions based on protein sequence clustering in the *Aplysina aerophoba* microbiome

The proteins detected in *A*. *aerophoba* affiliated to Bacteria (1,345), were clustered at 95% amino acid sequence identity. This gave an indication of the abundant proteins as associated with a given bacterial species, based on the average amino acid identity in genomes of the same species reported previously^21^. Based on this analysis, we found a total of 33 clusters with more than 5 proteins (see Supplementary Table S6). On the basis of their BLAST-P annotation, representative sequences of the abundant clusters in the sponge matched those of predicted proteins from groups of known symbionts of sponges. This was the case for “*Candidatus* Synechococcus spongiarum”, *Entotheonella*, and Chloroflexi^14,22,23^. Using this protein similarity clustering approach, the most abundant function was a putative sigma factor antagonist (12 proteins) from a bacterium resembling an uncultured Acidobacterium (query coverage 93%, sequence identity 35%). The second most abundant group of proteins (8) matched a hypothetical protein sequence from an unclassified Gammaproteobacterium (identity 56%) previously identified in marine metagenomes. The protein groups matching proteins from *Entotheonella* were mostly “hypothetical”, having an identity match of approximately 70%. For *S*. *spongiarum*, the most abundant functions correspond to putative toxin removal (i.e. bleomycin hydrolases) or were related to the synthesis of ATP. The Chloroflexi-affiliated proteins corresponded to heat shock proteins Hsp60 (alternative 60 kDa chaperonin).

### Proteins related to nitrogen and sulfur metabolism in the metaproteome of *A*. *aerophoba*

From the 1,345 bacterial proteins uniquely found in the sponge, we performed a detailed analysis of the expressed proteins related with nitrogen and sulfur transformations. Thus, with respect to nitrogen cycling in the sponge microbiome we found nine putative nitrate oxidoreductases assigned to *Nitrospira*, one putative copper-containing nitrite reductase (NirK), and 4 putative nitric oxide reductase subunit NorQ proteins matching similar ones of Entotheonellaceae in *A*. *aerophoba* (78% identity) (Table 4). Furthermore, with respect to sulfur cycling proteins, the analysis showed three putative sulfane dehydrogenases (subunit SoxC) from an alphaproteobacterium in the genus *Roseovarius*, and 10 putative sulfurtransferases from Chloroflexi in *A*. *aerophoba* (Table 4). These proteins were consistently found across several of the sponge individuals tested (Table 4).

**Table 4:** Functional assignment (KEGG orthology) of proteins affiliated to Bacteria domain corresponding to Nitrogen and Sulfur cycles in the sponge *A*. *aerophoba*.

KO	Proteins	KO function [Best BLASTP hit taxon]	Individuals	Coverage	Identity	Evalue
K00370	4	NarG; nitrate oxidoreductase subunit alpha [*Nitrospira moscoviensis*]	16C, 17	100%	85%	0.0
K00371	5	NarH; nitrate oxidoreductase subunit beta [*Nitrospira moscoviensis*]	16C, 17	100%	94%	0.0
K00368	1	NirK; nitrite reductase (copper-containing, NO-forming) [*Pseudovibrio sp*. Ad37]	19B	98%	80%	8.00E-126
K04748	4	NorQ; nitric oxide reductase NorQ protein [*Candidatus* Entotheonella sp. TSY1]	16C, 17, 19A, 19B	100%	78%	1.00E-164
K17225	3	SoxC; sulfane dehydrogenase subunit C [*Roseovarius nanhaiticus*]	16C, 19A, 19B	97%	68%	0.0
K01011	10	TST; thiosulfate/3-mercaptopyruvate sulfurtransferase [Chloroflexi bacterium 13_1_40CM_4_68_4]	16C, 17, 19B	98%	67%	1.00E-136

Individual samples where proteins were found, query coverage, amino-acid identity, and E-value are shown. Numbers indicate abundance.

### Carbohydrate-active enzymes in the metaproteome of *A*. *aerophoba*

**Figure 5:**
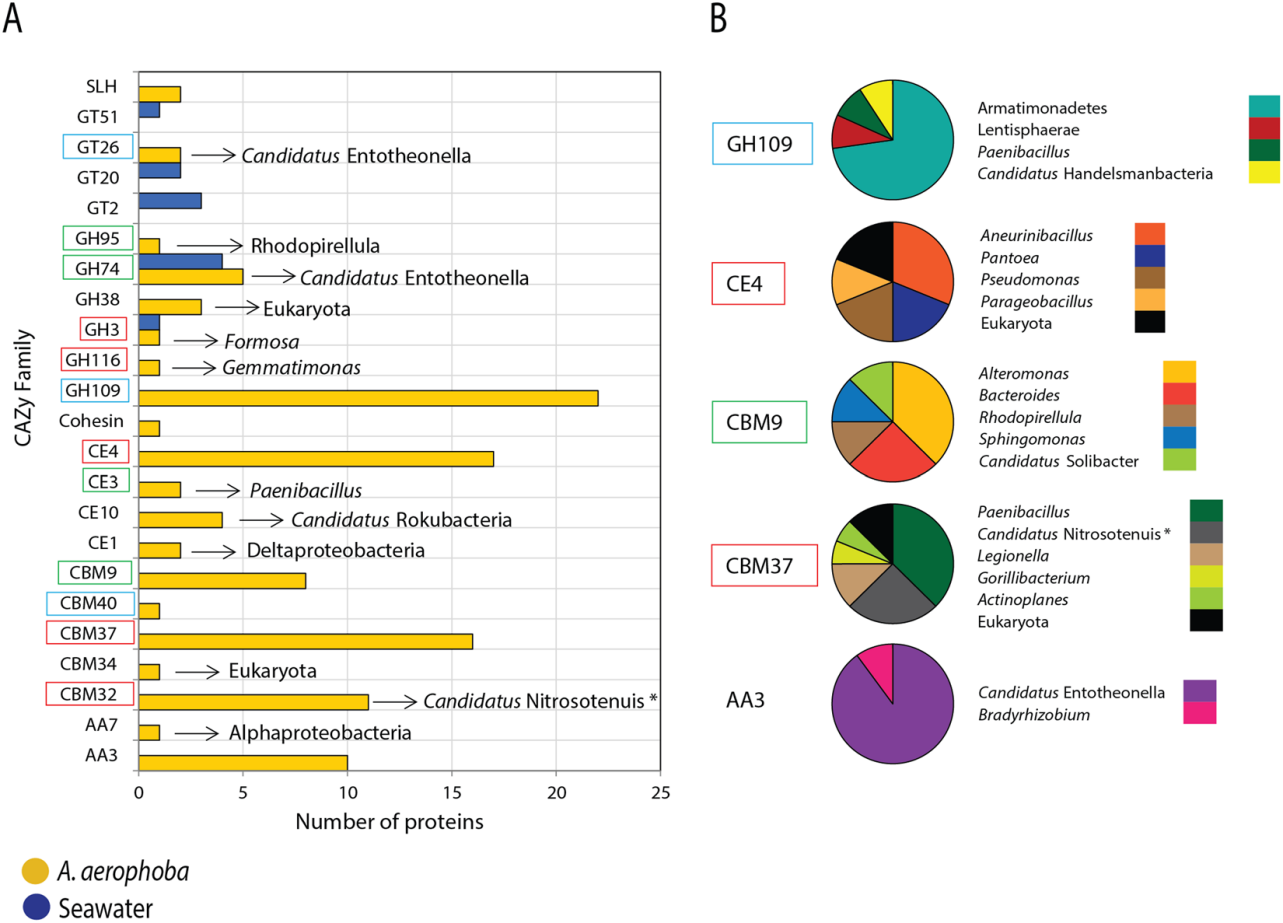
Carbohydrate-active enzymes in each habitat. (**A**) Number of proteins per habitat that were affiliated to CAZy families. Inside arrows pinpoint the taxonomic affiliation of the proteins within each family based on the best hit by BlastP (Supplementary Table S7). (**B**) Taxonomic affiliation of the proteins within the families GH109, CE4, CBM9, CBM37 and AA3 (oxidoreductases). Red, blue and green frame-squares represents the families that are probably involved in metabolism of chitin/N-acetylglucosamine; glycoproteins/glycolipids; and other complex polysacharides (e.g. xylan and xyloglucan), respectively. Asterisks represent archaeal taxa.

From the 2,987 unique proteins that were uniquely detected in the sponge dataset, a total of 111 (3.7%) was annotated as carbohydrate-active (CAZy) enzymes (see Supplementary Table S7). These proteins fell within 23 different CAZy families (Fig. 5A). The most abundant families (more than four proteins) were GH109, CE4, CBM37, CBM32, AA3, CBM9 and GH74, and are always present between three to all six samples analyzed. Five of these 23 families are involved in the metabolism of chitin and N-acetylglucosamine (GH3, GH116, CE4, CBM37 and CBM32), three in the bioconversion of glycoproteins and glycolipids (GT26, GH109, and CBM40) and four in the degradation of other complex polysaccharides like xylan and/or xyloglucan (GH95, GH74, CE3, CBM9). In order to infer the microbial source of each CAZy family protein, all relevant proteins were analyzed by BLAST_P against the NCBI nr database (see Supplementary Table S7). Based on the best hit to a CAZy domain, the proteins from family GH109 (EC 3.2.1.49; α-N-acetylgalactosaminidase) were mostly affiliated to a hypothetical protein (accession number OIP04581.1) from an Armatimonadetes bacterium (16 proteins, Fig. 5B). In the case of family CE4 (polysaccharide deacetylases), 13 proteins were associated with four bacterial genera (*Aneurinibacillus*, *Pantoea*, *Pseudomonas* and *Parageobacillus*). In addition, eight proteins belonging to family CBM9 (binding domain associated with xylanases) were mostly affiliated to those predicted from species in the *Alteromonas* and *Bacteroides* genera. Moreover, proteins associated with similar ones from *Paenibacillus* were found within families GH109, CBM37 (binding domain associate with polysaccharide-degrading enzymes) and CE3. In contrast, proteins associated with those from Entotheonellaceae were found in the families GT26 (glycosyl transferases), GH74 (endo-xyloglucanses) and AA3 (glucose-methanol-choline oxidoreductases). Regarding the conversion of N-acetylglucosamine (chitin monomer), proteins within families CBM32 (binding domain associate with N-acetylglucosaminidases), GH116 and GH3 (β-N-acetylglucosaminidases) were affiliated with those from “*Candidatus* Nitrosotenuis” (Archaea domain), *Formosa* sp. and *Gemmatimonas* sp., respectively.

## Discussion

The present study is the first metaproteomics-based study that analyses actual activities in the *Aplysina aerophoba* microbiome and compares these to those in the surrounding seawater. We sequenced 19,673 peptide fragments and identified 3,226 proteins traceable (unique) to the sponge microbial metagenome, the largest described so far for a sponge species. Our protein extraction method has previously been used on oleaginous mixed microbial communities from biological wastewater treatment plants^9^, human fecal samples^24^ and freshwater planktonic microbial communities^24^, and has revealed it was capable of extracting proteins related to translation, ribosomal structure and biogenesis, protein turn- over, chaperones, energy production and conversion, and lipid transport and metabolism. This leaves the possibility that other processes are undersampled, and so we recommend that our data are considered with a cautionary note in mind.

Our approach focused on the set of orthologous proteins existing uniquely in the sponge (2,987), and particularly on the bacteria in the system. Our results for the taxa contributing the largest number of proteins are consistent with previous 16S rRNA gene amplicon studies that identified members of Acidobacteria, Actinobacteria, Chloroflexi, Cyanobacteria and Alpha-, Gamma- and Deltaproteobacteria in *Aplysina* species^2,25,26^. This pattern does not hold for previously available metagenomic studies, where Proteobacteria, Firmicutes and Bacteroidetes represent the most abundant phyla^13^. In our study, we clearly identified proteins that were very likely produced by sponge-associated bacteria belonging to Cyanobacteria and the candidate phyla Tectomicrobia and Poribacteria^2,10,11,27,28^. However, we acknowledge that the overall amino acid identities (<70%) were indicative of the presence of organisms that are related to these taxa rather than being exact representatives of known, or sequenced members of these.

Regarding the functional profiles, high expression of high-affinity uptake systems was found, such as periplasmic substrate-binding domains associated with ABC transporters (in particular for amino acids) (Table 2), consistent with previous work^15^. In addition, five tripartite ATP-independent periplasmic (TRAP)-type transporters that are presumably specific to mannitol and chloroaromatic compounds, were detected. Chloroaromatic (more generally: haloaromatic) compounds are commonly found in marine sponges^29^, and numerous sponge-inhabiting bacteria are able to metabolize these. Such compounds might be released by sponge symbionts. For instance, all transporters of chloroaromatic compounds found exhibited similarity to proteins from *Thalassobaculum litoreum* DSM 18839. Interestingly, this chemoheterotrophic alphaproteobacterium, a nitrate reducer^30^, is one of the dominant OTUs in several intertidal and subtidal sponges^31^. Furthermore, we have found evidence for Chloroflexi expressing CO-dehydrogenase (CODH), potentially indicating that these microbes are involved in the chemolithoautotrophic utilization of CO that emerges in the system. This may well be linked to ATP production, providing a method for energy generation in these Chloroflexi.

The clustering of abundant functions and the LCA taxonomic analyses enabled us to confirm “*Candidatus* Synechococcus spongiarum” as a metabolically active symbiont of the sponge. This candidate species occurs in a variety of sponge species^32^, including *A*. *fulva*^26^ and *A*. *aerophoba*^14^. In addition, “*Candidatus* Synechococcus spongiarum” can be vertically transferred from parental *Chondrilla australiensis* to the next generation^33^. An overview of the protein set found by us (see Supplementary Table S4) revealed mainly proteins involved in amino acid transport, synthesis of pigments for photosynthesis. It is well established that sponges in shallow waters have dense populations of photosynthetic cyanobacteria, with photosynthate being translocated from these bacteria to the sponge^34^. In addition, “*Candidatus* Synechococcus spongiarum” had a large repertoire of bleomycin hydrolases. Given that genomes of particular Actinomycetes^35^ and Gammaproteobacteria (*Alteromonas*)^36^ encode proteins involved in the biosynthesis of bleomycin-like antibiotics, such organisms may abound in the microbiome studied; possibly, *Candidatus* Synechococcus spongiarum symbionts degrade such antibiotics in an anti-toxification process beneficial to itself and the host.

The presence of a Candidatus Entotheonella-like microbe in *A. aerophoba* has been suggested before on the basis of the 16S rRNA gene studies. However, the bacteria known as filamentous were not observed by using transmission electron microscopy^37^. We here provide evidence for the tenet that a Tectomicrobium, related to *Entotheonella* is highly active in *A. aerophoba* (Figs 2–4). Four *Entotheonella* genomes derived from metagenomes are available from the sponge *Theonella swinhoei* from the Japan Sea (Wilson *et al*.^20^) and the South Chinese Sea^38^. It has been shown that *Entotheonella sensu stricto* contain the genes for an aerobic, heterotrophic lifestyle and hence it may be capable of utilizing carbon sources such as chitin, N-acetylglucosamine, maltose and maltodextrin. We provide the first protein expression-based evidence for anaerobiosis features predicted for bacteria in Tectomictrobia bacteria. Entotheonella are known to possess *Nar*, *Nir*, *Nor* and *Nos* gene clusters^38^. With the expression evidence for norQ genes obtained in our study, it is likely that the tectomicrobium is capable of at least one step of anaerobic respiration through denitrification, i.e. the production of nitrous oxide from nitric oxide (NO). Putative nitric oxide reductase subunits (NorQ and NorD) have been detected previously in metagenomics, but not in proteomic, data sets for *C*. *concentrica*^15^, and so we add expression evidence to the previously-identified potential of conversion of nitric to nitrous oxide in a sponge. Another possibility is that NO plays an important role in the initiation of symbiotic colonization in the sponge tissue and *“Entotheonella”* is using a pathway that involves *norQ* to survive under high concentrations of NO in the host tissue. It is known that NO is produced as a defense response of animal cells to bacterial invaders, and nitric oxide synthases (NOS) result in high levels of NO during tissue colonization of the squid *Euprymna scolopes* by *Vibrio fischeri*^39^. *V. fischeri* effectively colonizes and irreversibly attenuates NO signals in the host tissues. A similar mechanism may be taking place between the sponge *A. aerophoba* and this Tectomicrobium during host colonization.

Microbially-mediated sulfur metabolism has been detected in sponges^17,18,40^. Both sulfate-reducing and sulfur-oxidizing microorganisms have been described, with sulfur-oxidizing bacteria (SOB) presumably oxidizing the reduced sulfur compounds generated by sulfate-reducing bacteria (SRB). *Thioalkalivibrio* sp. HK1 (Chromatiales) putatively oxidizes sulfide or sulfite through the reverse sulfate reduction pathway, based on the presence of the *soxABXYZ* and *dsrAB* genes, and the lack of *soxCD* genes^40^. Phototrophic anaerobic sulfur-oxidizing bacteria are mainly mesophilic and have been found previously in genera of the family Rhodobacteraceae, such as *Rhodobacter* and *Rhodovulum*^41^. Sulfite is also oxidized without SoxCD and studies have shown that in this case the cytochrome C reduction rate is decreased to 25%^42^. We found three sulfane dehydrogenases (subunit SoxC) from the peptide dataset matching *Roseovarius* (Rhodobacteraceae). The rest of the Sox pathway, namely SoxACXYZ, with the exception of SoxB, were found within the set of predicted proteins from the metagenomics assembly used in this work. The protein SoxC has been designated a sulfur dehydrogenase based on the phenotype displayed by a mutant with an in-frame deletion in the corresponding gene, which was not able to grow lithotrophically with thiosulfate but was still able to oxidize thiosulfate, although at a low rate^43^. Thus, we suggest that *Roseovarius* may be able to grow lithotrophically with thiosulfate as the electron donor. We cannot, by means of our approach, test the functionality of these or other identified genes, but based on their rather consistent appearance across samples (Table 4), and their absence in the seawater dataset, they appear essential to the system. Based on the absence of expression evidence of the accompanying subunits for known complex formation that include either NorQ or SoxC proteins, it may be that other subunits were unstable, produced less abundantly, or missed due to peptide sequencing depth. Given that sponges are relatively understudied, it is also possible that the actual function of these proteins in the sponge differs from those that have been described in other systems, for instance that other proteins fulfil the respective roles.

Moreover, we found evidence for the presence of a thiosulfate sulfur transferase (TST) [EC:2.8.1.1], also known as rhodanase, assigned to a *Chloroflexus*-like organism. This enzyme mediates the conversion of thiosulfate to sulfite and thiocyanate. It has been well described in eukaryotes^44^, where cyanide detoxification is an essential function. In prokaryotes, TST is known to occur in numerous taxa: chemolithotrophic and photosynthetic bacteria and heterotrophs with aerobic or anaerobic metabolism^45^. Bacteria possessing TST activity cleave thiosulfate and use it as a sole sulfur source^46^. In addition, these proteins can participate in the metabolism of cysteine. Given that Chloroflexi may have a wide range of metabolic activities, including fermentation, anoxygenic photosynthesis, nitrite oxidation and reductive dehalogenation^47^, they might be involved in a plethora of activities including sulfur transformations. The messengers for the aforementioned enzyme have recently found to be highly abundant in the transcriptome of Poribacteria^48^. With respect to the finding of rhodanase in these bacteria, we cannot affirm its potential function. Given that it was found in key symbiotic lineages in the sponge, it clearly deserves further exploration.

The extracellular matrix of sponges is rich in proteoglycans, adhesive glycoproteins and other structural proteins (collagen and chitin)^49^. In addition, seawater filtered by the sponge results in the uptake of chitin and other carbon polymers^50^. Specifically, the high abundance of proteins of CAZy family GH109 shows that bacteria in *A*. *aerophoba* express proteins involved in the breaking of the N-acetyl galactosamine linkages in the glycoproteins and glycolipids that are possibly present in the extracellular sponge protein structures. Interestingly, genes for CAZy family GH109 proteins were abundant in poribacterial genomes retrieved from *A*. *aerophoba* in the Mediterranean sea^11^. Thus family-GH109 α-N-acetyl galactosaminidases may constitute important functional traits in marine sponges’ microbiomes. Based on our data, these enzymes might not be restricted to the Poribacteria, as they are expressed by members of putative Armatimonadetes and Firmicutes.

Chitin (polysaccharide of N-acetyl-D-glucosamine monomers) constitutes a major component of the exoskeleton of *A*. *aerophoba*^49^. We show the expression of a putative chitin deacetylase (CE4), i.e. an enzyme involved in the deacetylation of β-N-acetyl glucosamine (GH3 and GH116), and of proteins with binding domains related to those of chitin-degrading enzymes (e.g. CBM32 and CBM37)^51^. The potential of sponge microbiomes to degrade chitin has previously been indicated, on the basis of a high prevalence of exochitinase genes in sponge microbiome DNA^12^. The degradation of chitin by sponge-associated microbes enables these to recruit carbon and nitrogen sources. Here, we suggest that the conversion of chitin to smaller compounds, both from pumped seawater and the extracellular matrix of *A*. *aerophoba*, is carried out mainly by *Paenibacillus*, “*Candidatus* Nitrosotenuis”, *Legionella*, *Formosa* and *Gemmatimonas* species. Moreover, on the basis of our data, we suggest that the Tectomicrobium mediates the conversion of glucans through the expression of CAZy family GH74 endo-xyloglucanases. These proteins could act on the β-1,4-linkages of the glucans, allowing to degrade several poly- and oligosaccharides. Sponge glucans can occur in other glycoconjugates, which may be responsible for sponge self- versus non-self-recognition^52^. Remarkably, GH74 is the second most abundant CAZy family in Poribacteria genomes^11^.

## Methods

### Sample collection

Six sponge adults of *Aplysina aerophoba* were collected by SCUBA diving in June 2014 in Cala Montgó, Spain (42°06′ 52.6″N, 3°10′ 02.0″E) at 7.8 to 12.7 m depth. Individual samples were taken at locations between 3 to 15 meters apart from each other. They were collected using gloves, cutting approximately 5 cm^3^ of the sponge tissue and placing it in 50 mL sterile tubes. The *in situ* identification was done visually by Dr. Detmer Sipkema. Seawater was collected in the immediate vicinity of the sponges (at minimally 1 m of distance), in four locations, using 3 L sterile containers to compare putative sponge-associated bacterial proteins to bacterioplankton proteins. Immediately after collection, the sponge pieces were flash-frozen in liquid nitrogen and then stored at −80 °C. Seawater samples were filtered through a 0.22 μm polycarbonate filter (Millipore) and the filters were preserved at −80 °C.

### Biomolecule extraction

In order to facilitate subsequent ‘omics’ data integration and analysis, we followed the sequential biomolecule extraction method devised by Roume *et al*.^24^ (Section 2.2.1). Briefly, the methodology allows for reproducible isolation of high-quality genomic DNA, RNA, proteins, and polar and non-polar metabolites from single microbial community samples. Extracellular metabolites were initially cold (4 °C) solvent extracted by bead-beating (2 min at 20 Hz in a Retsch Mixer Mill MM400) in defined mixtures of polar (methanol and water) and non-polar solvents (chloroform). After removal of the respective metabolite fractions, the interphase pellet (along with the steel milling balls) was kept on ice for the subsequent total RNA (enriched in large RNA), genomic DNA, and sequential protein isolations and purifications using the AllPrep DNA/RNA/Protein Mini kit-based method (QA, Qiagen, Venlo, The Netherlands) according to the instructions of the manufacturer. Only the DNA and protein fractions were used for the present study.

### Metagenome sequencing, assembly and protein predictions

Six individual sponge samples and three seawater samples were sequenced in two lanes of Illumina HiSeq PE100 by the Research Group Genome Analytics (GMAK) at DSMZ (Braunschweig, Germany). The sequencing details of the metagenomic data accompanying the metaproteomics analysis in this study are provided in Supplementary Table S8. Paired-end reads from individual samples were assembled using Megahit (v1.0.3; parameters:–preset, meta-sensitive) and examined in order to estimate the number of predicted putatively-expressed proteins using Prodigal v2.0^53^. The *A*. *aerophoba* metagenomes assembled into 273 718 contigs >1 Kb and the number of predicted proteins was 782 998. The three seawater metagenomes assembled into 119 824 contigs >1 Kb. From these, 370 455 proteins were predicted. The proteins predicted from the metagenomics assemblies from each environment were used as a database for subsequent identification of the peptides and proteins produced from the mass-spectrometry datasets (below). We provide an assessment of the taxonomic origin of the metagenomes used for protein predictions on the basis of two approaches: based on reads matching 16S rRNA genes extracted directly from the metagenomes using SortMeRNA^54^, and from marker genes pulled out from the assembled contigs. To obtain a measure of relative abundance of Tectomicrobia, raw reads from each of the samples were mapped onto the consensus 16S rRNA marker gene sequence with stringent parameters (minimum sequence identity of 98%) using BBMap (https://jgi.doe.gov/data-and-tools/bbtools/). For this purpose, we used Phylosift^55^, which relies on the pplacer^56^ algorithm to place identified metagenomic reads and contigs onto reference phylogenies pre-built with FastTree^57^. Metagenome assemblies, predicted protein datasets, and information on sequenced peptides are available athttp://hdl.handle.net/10411/PIPXST.

### Detection of Tectomicrobia using CARD-FISH

CARD-FISH probes specific to each fragment of the probable Tectomicrobium 16 S rRNA gene were designed using ARB^58^. The sequence of probe 1 was 5′-AGCGACCCATTGTCTTGAC-3′ and the sequence of probe 2 was 5′-CTCTAGCCCCTCCCATCC-3′. Once the specificity of the probes was verified, they were checked for self-annealing using OligoCalc (http://biotools.nubic.northwestern.edu/OligoCalc.html). CARD-FISH experiments were performed as described in the Supplementary methods file. Slides were observed under a model BX41 microscope (Olympus, Tokyo, Japan) equipped with UV-filter to visualize DAPI staining (U-MNU2) and deep red filter (XF110-2). Images were captured with Infinity camera 3 mounted on the microscope and processed with Infinity software for Windows. Color editing was performed in Adobe® Photoshop software (San Jose, CA, United States). The color balance was the only parameter edited and the values changed were the same for all the analyzed slides. In pictures taken with the DAPI filter, the cyan was lowered to −50, the magenta to −20 and the blue was increased to +60. In the pictures taken with deep red filter, the red was increased to +100 and the magenta and yellow were lowered to −100. Images were merged using ImageJ (https://imagej.net/Welcome).

### Phylogenetic analysis for the 16S rRNA marker gene

The probable Tectomicrobia 16S rRNA consensus sequence was added to the set of sequences used to build the current Tectomicrobia phylogeny^20^, kindly provided by Jörn Piel. The sequences were aligned in the SILVA online aligner tool (https://www.arb-silva.de/aligner/). The alignment was trimmed using GBlocks server (http://molevol.cmima.csic.es/castresana/Gblocks_server.html). The phylogenetic tree was built using maximum likelihood as implemented in RAxML (Randomized Axelerated Maximum Likelihood), under the general time reversible (GTR) model of nucleotide substitution with the gamma model of rate heterogeneity, with 100 bootstrap iterations. The resulting tree was visualized using FigTree (http://tree.bio.ed.ac.uk/software/figtree/).

### Liquid chromatography-tandem mass spectrometry

Protein mixtures (10–50 μg in 20 µl) from the sponge and seawater samples were loaded on a NuPAGE 1 mm * 15 well 4–12%- Bis-Tris–gel (Invitrogen, Waltham, MA, USA). The 1D PAGE gel was run for 60 min at 60 V in order to concentrate the proteins and eliminate impurities. The gel was washed with several changes of water and stained overnight with Coomassie blue (G250, Biorad, Hercules, CA, USA). Each lane was cut as a single band and destained in 50 mM ammonium hydrogen carbonate/acetonitrile 1:1 and washed twice with 50 mM ammonium bicarbonate. Proteins were reduced (10 mM DTT in 50 mM ammonium bicarbonate) for 30 min at 56 °C, cooled to room temperature and alkylated (55 mM IAA, iodoacetamide, in 50 mM ammonium bicarbonate) for 30 min in the dark at room temperature. Gel pieces were washed in 50 mM ammonium hydrogen carbonate/acetonitrile 1:1 for 15 min and covered with acetonitrile until they shrank. Acetonitrile was removed and gel particles dried. In-gel digestion was performed by adding 10 ng/μL trypsin in 50 mM ammonium carbonate to cover the bands at 37 °C overnight.

Tryptic peptides were extracted twice by adding 5 μL 1% aqueous trifluoroacetic acid and sonication for 5 min. Chromatography of the extracted tryptic peptides was performed with the Ultimate 3000 HPLC system (Thermo Fisher Scientific, Waltham, MA, USA) coupled online to a Q-Exactive-Plus mass spectrometer with a NanoFlex source (Thermo Fisher Scientific, Waltham, MA, USA) equipped with a stainless steel emitter. Tryptic digests were loaded onto a 5 mm × 300 μm i.d. trapping micro column packed with PepMAP100 5 μm particles (Dionex, Waltham, MA, USA) in 0.1% formic acid at the flow rate of 20 μL/min. After loading and washing for 3 min, peptides were back-flush eluted onto a 50 cm × 75 μm i.d. nanocolumn, packed with Acclaim C18 PepMAP100 2 μm particles (Dionex, Waltham, MA, USA). The following mobile phase gradient was delivered at the flow rate of 300 nL/min: 3–50% of solvent B in 90 min; 50–80% B in 0.5 min; 90% B during 9.5 min, and back to 3% B in 1 min and held at 3% A for 14 min. Solvent A was 100:0 H_2_O/acetonitrile with 0.1% (v/v) formic acid and solvent B was 0:100 H_2_O/acetonitrile with 0.1% (v/v) formic acid. Mass spectrometry data were acquired using a data-dependent top-12 method dynamically choosing the most abundant not-yet-sequenced precursor ions from the survey scans (300–1650 Th) with a dynamic exclusion of 20 seconds. Sequencing was performed via higher energy collisional dissociation fragmentation with a target value of 1e5 ions determined with predictive automatic gain control. Isolation of precursors was performed with a window of 1.6. Survey scans were acquired at a resolution of 70,000 at m/z 200. Resolution for HCD spectra was set to 17,500 at m/z 200 with a maximum ion injection time of 50 ms. The normalized collision energy was set at 27. Furthermore, the S-lens RF level was set at 60 and the capillary temperature was set at 250 °C. Precursor ions with single, unassigned, or six and higher charge states were excluded from fragmentation selection.

### Data processing and peptide identification

The mass spectrometry datasets were produced for four sponge samples (plus two technical replicates for two individuals) and four seawater samples (Table 1). These were analyzed for peptide and protein identification using PEAKS software v8.0^59^. Database searches were carried out against the total metagenome-derived predicted proteins (described above), including both sponge and seawater proteins. The search parameters were defined as follows: (1) the protein-digesting enzyme was set to trypsin, (2) false discovery rate (FDR) ≤0.1%, (3) fragment mass error tolerance 0.02 Da, (4) discarded peptides >10 ppm mass error, (5) retained proteins with ≥2 unique peptide matches, (6) tolerated up to 2 missed cleavages, and (7) detected as a fixed modification carbamidomethylation (57) and variable modifications (oxidation-methionine, 16)^60^.

### Detection and analysis of unique proteins in sponge and seawater samples

To determine homologies between the proteins we used the OrthoVenn server^19^, which relies on an all-against-all similarity search using UBLAST (v7.0.1090)^61^ and a subsequent identification of putative orthology using orthAgogue (v1.0.3)^62^. This analysis allowed us to establish the unique orthologous clusters and singletons present in each habitat. For the proteins exclusively found in the sponge we conducted a deeper taxonomic and functional annotations using BLASTP^63^ against the NCBI nr database (downloaded February 2017) with a threshold e-value of 10^−5^. For taxonomy, we calculated the lowest common ancestor (LCA) algorithm implemented within MEGAN (v5.0)^64^ for the top 50 hits for each annotated protein. In addition, the unique proteins in the sponge were functionally classified using the BlastKOALA platform^65^. For proteins taxonomically assigned to the bacterial domain in the sponge datasets, we counted the total number of fragmentation spectra that map to their constitutive peptides or spectral count^66^ (SC), thereby generating an abundance estimate at the phylum level. Proteins with <10 spectra were labelled as ‘low’, between 11–40 spectra as ‘medium’ and >40 spectra as ‘high’ in abundance.

In order to obtain semi-quantitative data, we clustered the proteins affiliated to Bacteria by LCA using CD-HIT^67^ with a threshold of 95% amino acid identity. Clusters composed of more than five protein sequences were retrieved and denoted as “abundant”, as described in^60^. One representative protein, chosen at random, for each “abundant” cluster was annotated by BLASTp against the NCBI nr database^63^. The most frequent taxonomic assignment between the top 10 hits was used for each annotated protein. Additionally, the proteins detected unique to each habitat were affiliated with carbohydrate-active (CAZy) enzymes using the webserver dbCAN^68^ and then manually annotated by BLASTp against NCBI nr database.

## Electronic supplementary material

Supplementary Material
