# Supplementary material for: Expressed protein profile of a Tectomicrobium and other microbial symbionts in the marine sponge Aplysina aerophoba as evidenced by metaproteomics
Source: Sci Rep. 2018 Aug 7;8:11795. doi: 10.1038/s41598-018-30134-0 (PMC6081418; doi:10.1038/s41598-018-30134-0)
Supplement: Supplementary file 1 — Supplementary Material [file 41598_2018_30134_MOESM1_ESM.pdf]

Supplementary Material

Title: "Expressed protein profile of a Tectomicrobium and other microbial symbionts in the marine sponge *Aplysina aerophoba* as evidenced by metaproteomics"

Authors: Maryam Chaib De Mares, Diego Javier Jiménez, Giorgia Palladino, Johanna Gutleben, Laura A. Lebrun, Emilie E.L. Muller, Paul Wilmes, Detmer Sipkema, Jan Dirk van Elsas

Supplementary Methods 1. Catalyzed reporter deposition fluorescence *in situ* hybridization (CARD-FISH)

Four *A. aerophoba* samples were fixed overnight using ethanol and xylene, and embedded in liquid paraffin. Sections 5 µm thick were cut with a microtome and dried overnight at 37 °C. Then, paraffin was removed using xylene and ethanol series (100% twice, 96%, 80%, 70%, 2 min each – the % indicated are v/v) at room temperature (RT). Peroxidases were inactivated incubating slides in 0.2 M HCl (RT, 12 min), followed by 20 mM Tris-HCl (RT, 10 min). Tissue sections were first permeabilized with 0.5 µg/mL Proteinase K and incubated at 37 °C for 5 min. Slides were washed in 20 mM Tris-HCl for 10 min at RT. For the second permeabilization slides were dipped in low melting agarose 0.1% w/v (RT, 10 min). Tissue sections were covered and incubated with lysozyme 10 mg/mL in 0.05 M Ethylenediaminetetraacetic acid (EDTA) and 0.1 M Tris-HCl at 37 °C for 1 h, then washed. Samples were dehydrated using an ethanol series (50%, 80%, 100% RT, 3 min each – the % indicated are v/v) and air dried. Tissue sections were subsequently covered with hybridization buffer (0.9 M NaCl, 0.02 M Tris-HCl, 0.02 M (sodium dodecyl sulfate) SDS, 55% v/v formamide, 10% w/v dextran sulfate and 1% v/v blocking solution [0.1 maleic acid buffer M, 0.15 M NaCl, 10% w/v blocking reagent]) containing probe at 1/20 dilution of working solution (50 ng/µL) and incubated at 35 °C for 3 h. Then, slides were washed in prewarmed washing buffer (0.005 M EDTA, 0.02 M Tris-HCl, 0.01 M SDS and 0.013 NaCl M) (35 °C, 15 min), and fast dried at RT. At this point slides can be stored in the dark at +4 °C, if needed.

For amplification, slides were incubated in 1X (Phosphate-buffered saline) PBS at RT for 15 min. Then, tissue sections were covered with amplification substrate mix (1/200 dilution of 100X tyramide-Alexa647 conjugate in amplification substrate [1/100 dilution of freshly prepared H_2O_2 solution – 0.15% v/v H_2O_2 in 1X PBS – in amplification buffer – 1X PBS, 0.1% w/v blocking reagent, 2 M NaCl and 10% w/v dextran sulfate]). Slides were then incubated in humid chambers at 35 °C for 20 min. Slides were washed in 1X PBS at RT for 15 min, followed by milliQ water (RT, 1 min – the milliQ water is obtained using the Arium® pro ultrapure water systems from Sartorius, Göttingen, Germany) and air dried. Finally, slides were counterstained with mounting medium (Vectashield) supplemented with DAPI (0.5 mg/mL). At this point slides can be stored in the dark at -20 °C, if needed.

Supplementary figures

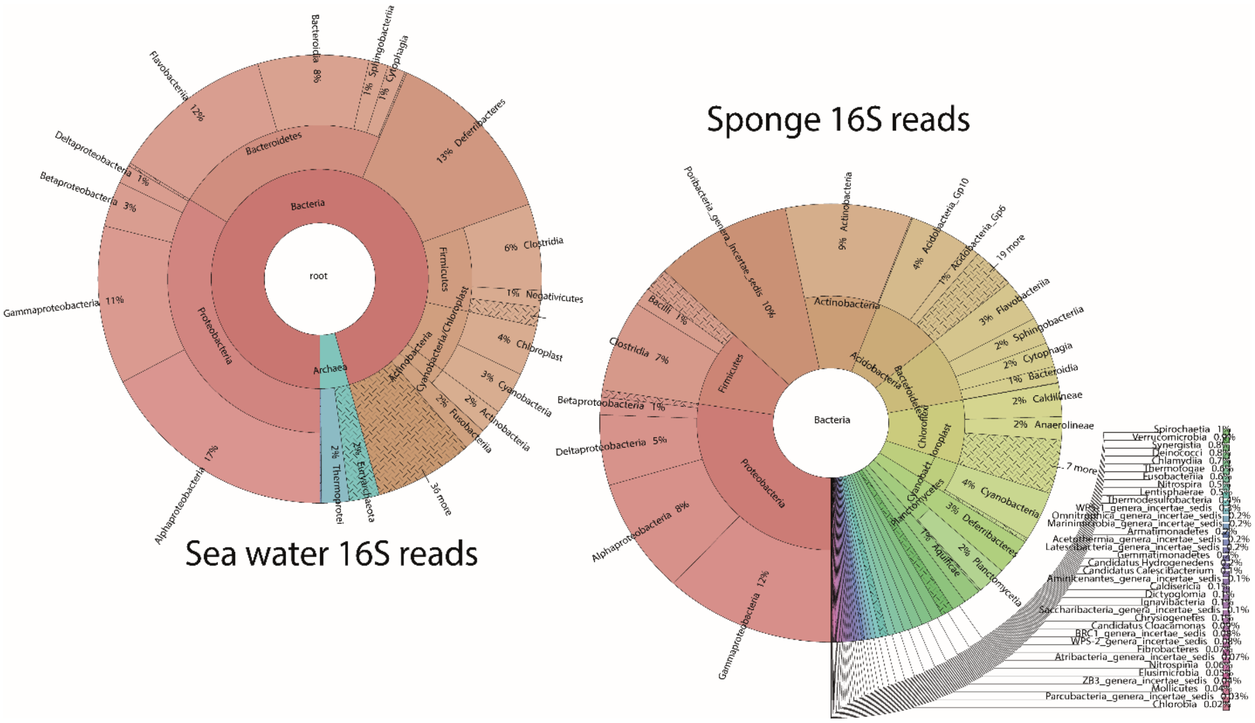

Supplementary Figure 1. Taxonomic assignments of metagenomic reads for sponge and seawater samples based on identified 16S rRNA marker genes.

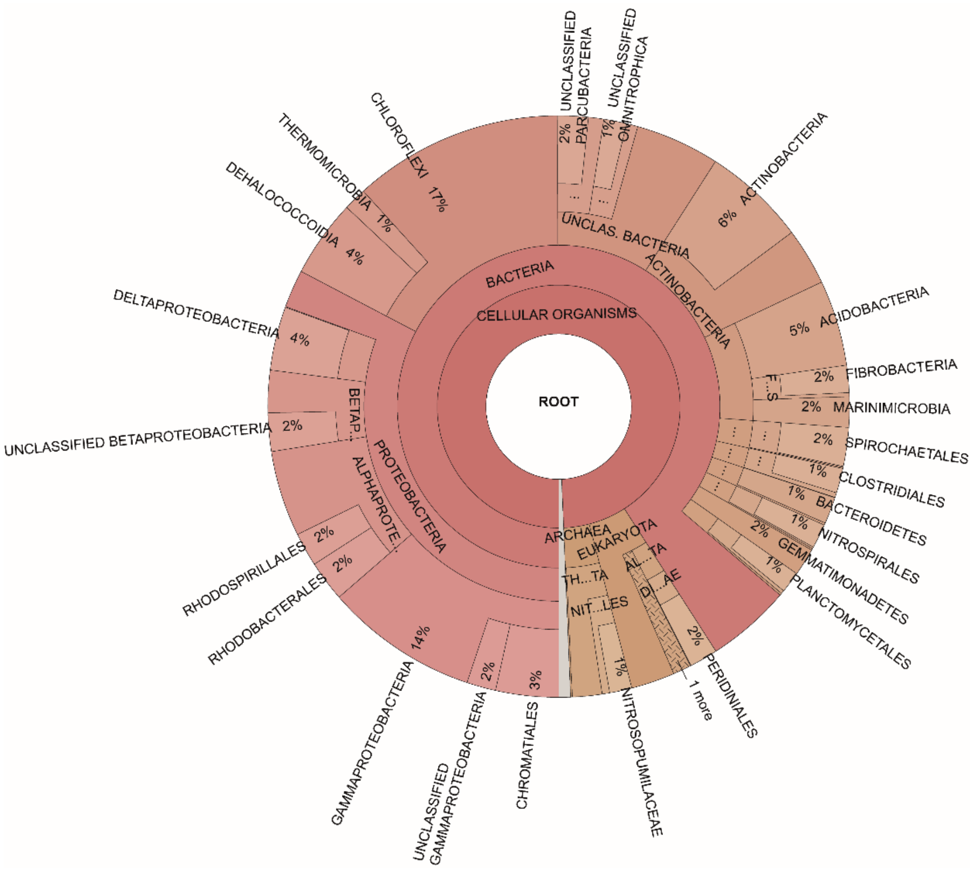

Supplementary figure 2. Taxonomic assignments based on phylogenetic markers present in assembled contigs of sponge samples using Phylosift (Darling *et al*. 2014).

Supplementary tables

Supplementary table S1. Abundance of spectral counts (sc) in bacterial phyla identified in sponge samples. "low": sc < 10, "medium": 10 < sc < 40, "high": sc > 40.

Phylum/abundance category	16B			16C			17			19A			19B			25			Overall		
	low	medium	high	low	medium	high	low	medium	high	low	medium	high	low	medium	high	low	medium	high	low	medium	high
**Acidobacteria**	11	3	0	13	1	0	9	2	0	5	0	0	9	3	0	1	2	0	48	11	0
**Actinobacteria**	14	3	3	43	3	0	21	5	1	29	2	0	24	2	0	7	3	1	138	18	5
**Alphaproteobacteria**	15	2	0	142	21	0	73	6	0	164	31	0	149	25	0	41	2	0	584	87	0
**Aquificae**	1	0	0	0	0	0	1	1	0	0	0	0	0	0	0	1	0	0	3	1	0
**Armatimonadetes**	1	0	0	0	0	0	1	0	0	4	0	0	2	0	0	0	0	0	8	0	0
**Bacteroidetes**	9	2	1	12	1	1	11	1	1	12	1	0	11	0	0	5	1	0	60	6	3
**Betaproteobacteria**	6	1	1	19	2	0	15	2	0	23	6	1	29	8	0	8	1	0	100	20	2
**Chlamydiae**	0	0	0	0	0	0	0	0	0	1	0	0	1	0	0	0	0	0	2	0	0
**Chlorobi**	5	2	0	1	1	1	4	0	1	8	0	0	3	0	0	3	0	1	24	3	3
**Chloroflexi**	12	1	0	28	1	0	10	5	0	25	3	0	34	2	0	7	2	0	116	14	0
**Chrysiogenetes**	1	0	0	0	0	0	1	0	0	0	0	0	0	0	0	1	0	0	3	0	0
**Cyanobacteria**	40	12	1	26	2	0	21	4	0	39	3	0	23	2	0	14	5	0	163	28	1
**Deferribacteres**	0	0	0	0	0	0	0	0	0	3	0	0	1	0	0	0	0	0	4	0	0
**Deinococcus-Thermus**	1	0	0	0	1	0	3	0	0	4	0	0	2	0	0	0	0	0	10	1	0
**Deltaproteobacteria**	14	1	0	14	1	0	8	2	0	15	2	0	13	2	0	4	1	0	68	9	0
**Dictyoglomi**	0	0	0	0	0	0	0	0	0	9	0	0	3	0	0	0	0	0	12	0	0
**Epsilonproteobacteria**	0	0	0	1	0	0	0	0	0	2	0	0	1	0	0	0	0	0	4	0	0
**Firmicutes**	22	8	2	38	1	0	18	5	0	38	2	0	36	1	0	9	4	0	161	21	2
**Gammaproteobacteria**	33	2	1	45	5	0	32	2	1	52	6	0	41	5	0	25	2	0	228	22	2
**Gemmatimonadetes**	3	0	0	3	0	0	2	1	0	2	0	0	0	0	0	0	0	0	10	1	0
**Nitrospirae**	2	0	0	3	2	0	3	1	0	0	2	0	0	1	1	1	0	0	9	6	1
**Other**	1	0	0	4	1	0	4	0	0	6	0	0	4	3	0	0	0	0	19	4	0
**Planctomycetes**	1	1	0	11	0	1	5	1	0	7	2	0	8	0	1	2	0	0	34	4	2
**Spirochaetes**	0	0	0	7	1	0	1	0	0	0	0	0	6	0	0	1	0	0	15	1	0
**Synergistetes**	0	0	0	2	0	0	1	0	0	1	0	0	2	1	0	0	0	0	6	1	0
**Thermotogae**	1	0	0	1	0	0	1	0	0	3	0	0	1	0	0	0	0	0	7	0	0

**Unclassified Terrabacteria group**	0	0	0	3	0	0	5	0	0	4	1	0	6	1	0	0	0	0	18	2	0
**Verrucomicrobia**	2	0	0	2	0	0	2	0	0	1	0	0	2	0	0	0	0	0	9	0	0

Supplementary table S2. Partial 16S rRNA genes from metagenome assembly matching Tectomicrobia.

sequence_ID	sequence_score	identity	quality	startpos	stoppos	Lowest_common_aancestor_taxonomy_(lca_tax)_GreenGenes	lca_tax_SILVA
k99_396	0.98707	98.3871	98	27658	43289	k__Bacteria;p__Proteobacteria;c__Deltaproteobacteria;o__[Entotheonellales];f__[Entotheonellaceae];	Bacteria;Tectomicrobia;
k99_337	0.995335	95.1496	99	887	29678	k__Bacteria;p__Proteobacteria;c__Deltaproteobacteria;o__[Entotheonellales];f__[Entotheonellaceae];	Bacteria;Tectomicrobia;

Supplementary table S3. Number of total bacterial proteins assigned to each KEGG Orthology (KO) category, with an indication of function, its hierarchical classification, and protein counts from each replicate.

KO	Protein name	Protein counts							Hierarchical classification
		Total Proteins	16B	16C	17	19A	19B	25	
K02035	ABC.PE.S; peptide/nickel transport system substrate-binding protein	140	3	25	11	56	39	6	Systems of aminoacids, sugars and ions transport
K01999	LivK; branched-chain amino acid transport system substrate-binding protein	82	3	22	10	25	20	2	Systems of aminoacids, sugars and ions transport
K04077	GroEL; chaperonin GroEL	58	14	4	12	8	9	11	Chaperonin GroEL and chaperone DnaK
K09969	AapJ; general L-amino acid transport system substrate-binding protein	37	2	7	6	6	11	5	Systems of aminoacids, sugars and ions transport
K02055	ABC.SP.S; putative spermidine/putrescine transport system substrate-binding protein	37	0	8	3	13	12	1	Systems of aminoacids, sugars and ions transport
K02051	ABC.SN.S; NitT/TauT family transport system substrate-binding protein	34	1	8	6	8	9	2	Systems of aminoacids, sugars and ions transport
K02027	ABC.MS.S; multiple sugar transport system substrate-binding protein	25	2	4	1	10	7	1	Systems of aminoacids, sugars and ions transport
K07080	uncharacterized protein	22	0	7	2	5	8	0	uncharacterized proteins
K02030	ABC.PA.S; polar amino acid transport system substrate-binding protein	20	0	6	0	7	7	0	Systems of aminoacids, sugars and ions transport
K01455	Formamidase [EC:3.5.1.49]	17	0	6	1	6	4	0	Nitrogen metabolism
K01053	Gluconolactonase [EC:3.1.1.17]	15	0	5	2	5	2	1	Carbon metabolism
K00114	ExaA; alcohol dehydrogenase (cytochrome c) [EC:1.1.2.8]	14	0	3	2	3	3	3	Carbon metabolism
K03530	HupB; DNA-binding protein HU-beta	14	11	0	1	0	0	2	Replication and repair
K03520	CoxL; carbon-monoxide dehydrogenase large subunit [EC:1.2.7.4]	14	11	0	1	0	0	2	Xenobiotics biodegradation and metabolism
K00031	IDH1; isocitrate dehydrogenase [EC:1.1.1.42]	13	1	1	1	2	8	0	Carbon metabolism
K05377	CpeB; phycoerythrin beta chain	12	2	2	2	2	2	2	Syntesis of pigments and photosynthesis
K01011	TST; thiosulfate/3-mercaptopyruvate sulfurtransferase [EC:2.8.1.1 2.8.1.2]	12	0	8	1	0	3	0	Sulfur metabolism
K05376	CpeA; phycoerythrin alpha chain	11	2	2	2	2	1	2	Syntesis of pigments and photosynthesis

K00024	Mdh; malate dehydrogenase [EC:1.1.1.37]	10	2	1	0	4	1	2	Carbon metabolism
K03519	CoxM; carbon-monoxide dehydrogenase medium subunit [EC:1.2.7.4]	10	1	6	0	1	2	0	Xenobiotics biodegradation and metabolism
K01915	GlnA; glutamine synthetase [EC:6.3.1.2]	9	2	3	2	1	1	0	Nitrogen metabolism
K02058	ABC.SS.S; simple sugar transport system substrate-binding protein	9	1	3	1	2	2	0	Systems of aminoacids, sugars and ions transport
K00962	Pnp; polyribonucleotide nucleotidyltransferase [EC:2.7.7.8]	9	1	2	1	3	2	0	Amino acid and nucleotide metabolism
K01061	Carboxymethylenebutenolidase [EC:3.1.1.45]	9	0	2	1	1	3	2	Xenobiotics biodegradation and metabolism
K00123	FdoG; formate dehydrogenase major subunit [EC:1.2.1.2]	9	0	2	2	3	2	0	Carbon metabolism
K02945	RP-S1; small subunit ribosomal protein S1	8	0	5	2	0	1	0	Ribosomal Proteins
K11069	PotD; spermidine/putrescine transport system substrate-binding protein	8	0	1	1	3	3	0	Systems of aminoacids, sugars and ions transport
K17285	SELENBP1; selenium-binding protein 1	8	0	1	2	3	2	0	Exosome
K02285	CpcB; phycocyanin beta chain	8	2	2	2	2	0	0	Syntesis of pigments and photosynthesis
K01652	Acetolactate synthase I/II/III large subunit [EC:2.2.1.6]	7	1	1	1	1	2	1	Carbon metabolism
K01251	Adenosylhomocysteinase [EC:3.3.1.1]	7	0	0	2	2	3	0	Amino acid and nucleotide metabolism
K03671	TrxA; thioredoxin 1	7	7	0	0	0	0	0	NOD-like receptor signaling pathway
K11930	TorT; periplasmic protein TorT	7	0	2	0	3	2	0	Systems of aminoacids, sugars and ions transport
K11959	UrtA; urea transport system substrate-binding protein	7	0	2	0	3	2	0	Systems of aminoacids, sugars and ions transport
K07303	IorB; isoquinoline 1-oxidoreductase subunit beta [EC:1.3.99.16]	7	0	1	1	2	2	1	Oxidoreductases
K02002	ProX; glycine betaine/proline transport system substrate-binding protein	6	1	1	1	3	0	0	Systems of aminoacids, sugars and ions transport
K11177	YagR; xanthine dehydrogenase YagR molybdenum-binding subunit [EC:1.17.1.4]	6	0	0	1	2	3	0	Amino acid and nucleotide metabolism
K00117	Gcd; quinoprotein glucose dehydrogenase [EC:1.1.5.2]	6	0	6	0	0	0	0	Carbon metabolism
K02031	ABC.PE.A; peptide/nickel transport system ATP-binding protein	6	0	3	0	0	3	0	Systems of aminoacids, sugars and ions transport
K02358	Tuf; elongation factor Tu	6	0	2	1	2	1	0	Translation factors
K02557	MotB; chemotaxis protein MotB	6	2	0	3	0	0	1	Bacterial chemotaxis
K13796	CobZ; tricarballylate dehydrogenase	5	0	0	3	2	0	0	Carbon metabolism
K01810	GPI; glucose-6-phosphate isomerase [EC:5.3.1.9]	5	0	1	2	1	1	0	Carbon metabolism
K03336	IolD; 3D-(3,5/4)-trihydroxycyclohexane-1,2-dione acylhydrolase (decyclizing) [EC:3.7.1.22]	5	0	0	0	0	5	0	Carbon metabolism
K00371	NarH; nitrate reductase / nitrite oxidoreductase, beta subunit [EC:1.7.5.1 1.7.99.4]	5	0	1	1	1	1	1	Nitrogen metabolism
K05379	CpeD; phycoerythrin-associated linker protein	5	1	1	1	1	1	0	Syntesis of pigments and photosynthesis
K10439	RbsB; ribose transport system substrate-binding protein	5	0	3	0	1	1	0	Systems of aminoacids, sugars and ions transport
K04043	Molecular chaperone DnaK	5	1	0	1	3	0	0	Chaperonin GroEL and chaperone DnaK

K00052	LeuB; 3-isopropylmalate dehydrogenase [EC:1.1.1.85]	5	0	0	0	2	3	0	Carbon metabolism
K02012	AfuA; iron(III) transport system substrate-binding protein	5	0	0	0	3	2	0	Systems of aminoacids, sugars and ions transport
K03696	ATP-dependent Clp protease ATP-binding subunit ClpC	5	0	0	0	1	4	0	Heat shock proteins
K01679	Fumarate hydratase, class II [EC:4.2.1.2]	4	0	0	4	0	0	0	Carbon metabolism
K00370	NarG; nitrate reductase / nitrite oxidoreductase, alpha subunit [EC:1.7.5.1 1.7.99.4]	4	0	1	1	1	1	0	Nitrogen metabolism
K02986	RP-S4; small subunit ribosomal protein S4	4	0	1	0	0	3	0	Ribosomal Proteins
K02284	CpcA; phycocyanin alpha chain	4	2	0	0	0	0	2	Syntesis of pigments and photosynthesis
K02286	CpcC; phycocyanin-associated rod linker protein	4	1	0	1	1	1	0	Syntesis of pigments and photosynthesis
K02112	ATPF1B; F-type H+-transporting ATPase subunit beta [EC:3.6.3.14]	4	0	0	0	3	0	1	Syntesis of pigments and photosynthesis
K00616	Transaldolase [EC:2.2.1.2]	4	0	0	0	1	2	1	Carbon metabolism
K17318	LplA; putative aldouronate transport system substrate-binding protein	3	0	1	0	1	1	0	Systems of aminoacids, sugars and ions transport
K00789	MetK; S-adenosylmethionine synthetase [EC:2.5.1.6]	3	0	0	0	0	3	0	Amino acid and nucleotide metabolism
K02433	GatA; aspartyl-tRNA(Asn)/glutamyl-tRNA(Gln) amidotransferase subunit A [EC:6.3.5.6 6.3.5.7]	3	0	0	0	3	0	0	Aminoacyl-tRNA biosynthesis
K00162	PDHB; pyruvate dehydrogenase E1 component beta subunit [EC:1.2.4.1]	3	0	0	0	1	2	0	Carbon metabolism
K14733	LimB; limonene 1,2-monooxygenase [EC:1.14.13.107]	3	0	1	0	2	0	0	Metabolism of terpenoids and polyketides
K17225	SoxC; sulfane dehydrogenase subunit SoxC	3	0	1	0	1	1	0	Sulfur metabolism
K02692	PsaD; photosystem I subunit II	3	1	0	1	1	0	0	Syntesis of pigments and photosynthesis
K02014	TC.FEV.OM; iron complex outermembrane recepter protein	3	1	0	2	0	0	0	Systems of aminoacids, sugars and ions transport
K02838	Frr; ribosome recycling factor	3	3	0	0	0	0	0	Translation factors
K01607	PcaC; 4-carboxymuconolactone decarboxylase [EC:4.1.1.44]	3	3	0	0	0	0	0	Xenobiotics biodegradation and metabolism
K01255	CARP; leucyl aminopeptidase [EC:3.4.11.1]	2	0	0	0	1	1	0	Amino acid and nucleotide metabolism
K02337	DPO3A1; DNA polymerase III subunit alpha [EC:2.7.7.7]	2	0	1	0	1	0	0	Amino acid and nucleotide metabolism
K01555	FAH; fumarylacetoacetase [EC:3.7.1.2]	2	0	0	0	0	2	0	Amino acid and nucleotide metabolism
K00383	GSR; glutathione reductase (NADPH) [EC:1.8.1.7]	2	0	1	0	1	0	0	Amino acid and nucleotide metabolism
K01003	BcpA; oxaloacetate decarboxylase [EC:4.1.1.3]	2	0	1	0	0	1	0	Carbon metabolism
K10713	Fae; 5,6,7,8-tetrahydromethanopterin hydro-lyase [EC:4.2.1.147]	2	0	0	0	2	0	0	Carbon metabolism
K00855	PRK; phosphoribulokinase [EC:2.7.1.19]	2	0	1	0	1	0	0	Carbon metabolism
K01601	RbcL; ribulose-bisphosphate carboxylase large chain [EC:4.1.1.39]	2	0	0	0	2	0	0	Carbon metabolism
K01356	LexA; repressor LexA [EC:3.4.21.88]	2	0	0	0	0	2	0	Drug resistance
K03386	PRDX2_4; peroxiredoxin (alkyl hydroperoxide reductase subunit C) [EC:1.11.1.15]	2	0	1	0	1	0	0	Oxidoreductases

K00518	SodN; nickel superoxide dismutase [EC:1.15.1.1]	2	1	0	0	0	1	0	Oxidoreductases
K02988	RP-S5; small subunit ribosomal protein S5	2	0	2	0	0	0	0	Ribosomal Proteins
K02092	ApcA; allophycocyanin alpha subunit	2	1	0	0	0	0	1	Syntesis of pigments and photosynthesis
K02044	PhnD; phosphonate transport system substrate-binding protein	2	0	0	0	1	1	0	Systems of aminoacids, sugars and ions transport
K03684	Rnd; ribonuclease D [EC:3.1.13.5]	2	0	0	0	0	0	2	Transfer RNA biogenesis
K01895	ACSS; acetyl-CoA synthetase [EC:6.2.1.1]	2	0	0	0	1	1	0	Carbon metabolism
K00284	Glutamate synthase (ferredoxin) [EC:1.4.7.1]	2	0	0	0	2	0	0	Carbon metabolism
K06215	Pyridoxal 5'-phosphate synthase PdxS subunit [EC:4.3.3.6]	2	0	0	0	0	2	0	Metabolism of cofactors and vitamins
K03555	DNA mismatch repair protein MutS	2	0	1	1	0	0	0	Replication and repair
K02109	ATPF0B; F-type H+-transporting ATPase subunit b	2	2	0	0	0	0	0	Syntesis of pigments and photosynthesis
K04749	RsbV; anti-sigma B factor antagonist	1	1	0	0	0	0	0	Transcription factors
K00108	BetA; choline dehydrogenase [EC:1.1.99.1]	1	0	1	0	0	0	0	Amino acid and nucleotide metabolism
K00821	ArgD; acetylornithine/N-succinyldiaminopimelate aminotransferase [EC:2.6.1.11 2.6.1.17]	1	0	1	0	0	0	0	Amino acid and nucleotide metabolism
K00059	FabG; 3-oxoacyl-[acyl-carrier protein] reductase [EC:1.1.1.100]	1	0	0	0	0	1	0	Fatty acid metabolism
K03856	AROA2; 3-deoxy-7-phosphoheptulonate synthase [EC:2.5.1.54]	1	0	0	0	0	1	0	Amino acid and nucleotide metabolism
K01620	LtaE; threonine aldolase [EC:4.1.2.48]	1	0	1	0	0	0	0	Amino acid and nucleotide metabolism
K00940	Ndk; nucleoside-diphosphate kinase [EC:2.7.4.6]	1	0	0	0	1	0	0	Amino acid and nucleotide metabolism
K01872	AARS; alanyl-tRNA synthetase [EC:6.1.1.7]	1	0	0	1	0	0	0	Aminoacyl-tRNA biosynthesis
K01887	RARS; arginyl-tRNA synthetase [EC:6.1.1.19]	1	0	0	1	0	0	0	Aminoacyl-tRNA biosynthesis
K15923	AXY8; alpha-L-fucosidase 2 [EC:3.2.1.51]	1	0	0	0	1	0	0	Carbon metabolism
K01684	DgoD; galactonate dehydratase [EC:4.2.1.6]	1	0	0	0	0	1	0	Carbon metabolism
K11779	FbiC; FO synthase [EC:2.5.1.77]	1	0	0	0	0	0	1	Carbon metabolism
K00135	GabD; succinate-semialdehyde dehydrogenase / glutarate-semialdehyde dehydrogenase [EC:1.2.1.16 1.2.1.79 1.2.1.20]	1	0	0	0	1	0	0	Carbon metabolism
K00042	GarR; 2-hydroxy-3-oxopropionate reductase [EC:1.1.1.60]	1	0	0	0	0	1	0	Carbon metabolism
K00104	GlcD; glycolate oxidase [EC:1.1.3.15]	1	0	1	0	0	0	0	Carbon metabolism
K00820	GlmS; glucosamine--fructose-6-phosphate aminotransferase (isomerizing) [EC:2.6.1.16]	1	0	0	0	1	0	0	Carbon metabolism
K00873	PK; pyruvate kinase [EC:2.7.1.40]	1	0	1	0	0	0	0	Carbon metabolism
K01006	PpdK; pyruvate, orthophosphate dikinase [EC:2.7.9.1]	1	0	1	0	0	0	0	Carbon metabolism
K00239	SdhA; succinate dehydrogenase / fumarate reductase, flavoprotein subunit [EC:1.3.5.1 1.3.5.4]	1	0	0	1	0	0	0	Carbon metabolism
K01805	XylA; xylose isomerase [EC:5.3.1.5]	1	0	0	0	1	0	0	Carbon metabolism

K19123	CasA; CRISPR system Cascade subunit CasA	1	0	0	0	0	0	1	CRISPR-Cas system
K08641	VanX; D-alanyl-D-alanine dipeptidase [EC:3.4.13.22]	1	1	0	0	0	0	0	Drug resistance
K00208	FabI; enoyl-[acyl-carrier protein] reductase I [EC:1.3.1.9 1.3.1.10]	1	0	0	0	1	0	0	Fatty acid metabolism
K03694	ClpA; ATP-dependent Clp protease ATP-binding subunit ClpA	1	0	0	1	0	0	0	Heat shock proteins
K01845	HemL; glutamate-1-semialdehyde 2,1-aminomutase [EC:5.4.3.8]	1	0	0	0	1	0	0	Metabolism of cofactors and vitamins
K00510	HMOX; heme oxygenase (biliverdin-producing) [EC:1.14.14.18]	1	1	0	0	0	0	0	Metabolism of cofactors and vitamins
K00053	IlvC; ketol-acid reductoisomerase [EC:1.1.1.86]	1	0	0	0	1	0	0	Metabolism of cofactors and vitamins
K03639	MoaA; GTP 3',8-cyclase [EC:4.1.99.22]	1	0	0	0	0	1	0	Metabolism of cofactors and vitamins
K00788	ThiE; thiamine-phosphate pyrophosphorylase [EC:2.5.1.3]	1	0	0	0	0	0	1	Metabolism of cofactors and vitamins
K00368	NirK; nitrite reductase (NO-forming) [EC:1.7.2.1]	1	0	0	0	0	1	0	Nitrogen metabolism
K03564	BCP; peroxiredoxin Q/BCP [EC:1.11.1.15]	1	0	0	0	0	1	0	Oxidoreductases
K01972	DNA ligase (NAD+) [EC:6.5.1.2]	1	0	0	1	0	0	0	Replication and repair
K02864	RP-L10; large subunit ribosomal protein L10	1	1	0	0	0	0	0	Ribosomal Proteins
K02867	RP-L11; large subunit ribosomal protein L11	1	1	0	0	0	0	0	Ribosomal Proteins
K02871	RP-L13; large subunit ribosomal protein L13	1	0	1	0	0	0	0	Ribosomal Proteins
K04751	GlnB; nitrogen regulatory protein P-II 1	1	1	0	0	0	0	0	Signal transduction
K02093	ApcB; allophycocyanin beta subunit	1	1	0	0	0	0	0	Syntesis of pigments and photosynthesis
K02694	PsaF; photosystem I subunit III	1	1	0	0	0	0	0	Syntesis of pigments and photosynthesis
K02719	PsbU; photosystem II PsbU protein	1	1	0	0	0	0	0	Syntesis of pigments and photosynthesis
K02720	PsbV; photosystem II cytochrome c550	1	1	0	0	0	0	0	Syntesis of pigments and photosynthesis
K02004	ABC.CD.P; putative ABC transport system permease protein	1	1	0	0	0	0	0	Systems of aminoacids, sugars and ions transport
K06147	ABCB-BAC; ATP-binding cassette, subfamily B, bacterial	1	0	0	1	0	0	0	Systems of aminoacids, sugars and ions transport
K10227	SmoE; sorbitol/mannitol transport system substrate-binding protein	1	0	0	0	1	0	0	Systems of aminoacids, sugars and ions transport
K15551	TauA; taurine transport system substrate-binding protein	1	0	1	0	0	0	0	Systems of aminoacids, sugars and ions transport
K05813	UgpB; sn-glycerol 3-phosphate transport system substrate-binding protein	1	0	0	0	1	0	0	Systems of aminoacids, sugars and ions transport
K03556	MalT; LuxR family transcriptional regulator, maltose regulon positive regulatory protein	1	0	0	0	0	1	0	Transcription factors
K02601	NusG; transcriptional antiterminator NusG	1	0	0	0	0	1	0	Transcription factors
K02357	Tsf; elongation factor Ts	1	1	0	0	0	0	0	Translation factors
K01055	PcaD; 3-oxoadipate enol-lactonase [EC:3.1.1.24]	1	1	0	0	0	0	0	Xenobiotics biodegradation and metabolism
K04748	NorQ; nitric oxide reductase NorQ protein	0	0	0	0	0	0	0	Nitrogen metabolism

K02510	HpaI; 4-hydroxy-2-oxoheptanedioate aldolase [EC:4.1.2.52]	0	0	0	0	0	0	0	Amino acid and nucleotide metabolism
K11532	GlpX-SEBP; fructose-1,6-bisphosphatase II / sedoheptulose-1,7-bisphosphatase [EC:3.1.3.11 3.1.3.37]	0	0	0	0	0	0	0	Carbon metabolism
K00008	SORD; L-iditol 2-dehydrogenase [EC:1.1.1.14]	0	0	0	0	0	0	0	Carbon metabolism
K12368	DppA; dipeptide transport system substrate-binding protein	0	0	0	0	0	0	0	Systems of aminoacids, sugars and ions transport
K10823	OppF; oligopeptide transport system ATP-binding protein	0	0	0	0	0	0	0	Systems of aminoacids, sugars and ions transport

Supplementary table S4. Number of proteins assigned to the genus *Synechococcus*, split based on KEGG Orthology (KO) category.

**KO**	**Function**	**Number of Proteins**
K05376	CpeA; phycoerythrin alpha chain	11
K05377	CpeB; phycoerythrin beta chain	6
K05379	CpeD; phycoerythrin-associated linker protein	5
K02284	CpcA; phycocyanin alpha chain	4
K02286	CpcC; phycocyanin-associated rod linker protein	4
K00962	Pnp; polyribonucleotide nucleotidyltransferase [EC:2.7.7.8]	3
K01915	GlnA; glutamine synthetase [EC:6.3.1.2]	3
K02692	PsaD; photosystem I subunit II	3
K02035	Peptide/nickel transport system substrate-binding protein	3
K11532	GlpX-SEBP; fructose-1,6-bisphosphatase II / sedoheptulose-1,7-bisphosphatase [EC:3.1.3.11 3.1.3.37]	2
K00855	PRK; phosphoribulokinase [EC:2.7.1.19]	2
K03386	PRDX2_4; peroxiredoxin (alkyl hydroperoxide reductase subunit C) [EC:1.11.1.15]	2
K00518	SodN; nickel superoxide dismutase [EC:1.15.1.1]	2
K02092	ApcA; allophycocyanin alpha subunit	2
K02285	CpcB; phycocyanin beta chain	2
K01999	LivK; branched-chain amino acid transport system substrate-binding protein	2
K00821	ArgD; acetylornithine/N-succinyldiaminopimelate aminotransferase [EC:2.6.1.11 2.6.1.17]	1
K00940	Ndk; nucleoside-diphosphate kinase [EC:2.7.4.6]	1
K01601	RbcL; ribulose-bisphosphate carboxylase large chain [EC:4.1.1.39]	1
K00208	FabI; enoyl-[acyl-carrier protein] reductase I [EC:1.3.1.9 1.3.1.10]	1
K00510	HMOX; heme oxygenase (biliverdin-producing) [EC:1.14.14.18]	1
K00053	IlvC; ketol-acid reductoisomerase [EC:1.1.1.86]	1

K03564	BCP; peroxiredoxin Q/BCP [EC:1.11.1.15]	1
K02864	RP-L10; large subunit ribosomal protein L10	1
K02093	ApcB; allophycocyanin beta subunit	1
K02109	ATPF0B; F-type H+-transporting ATPase subunit b	1
K02694	PsaF; photosystem I subunit III	1
K02719	PsbU; photosystem II PsbU protein	1
K02720	PsbV; photosystem II cytochrome c550	1
K02601	NusG; transcriptional antiterminator NusG	1

Supplementary table S5. Proteins assigned to *Candidatus* Methylomirabilis oxyfera and *Candidatus* Poribacteria.

ID protein	Replicate	Size aa	Best Blastp hit [taxa]	Score	Query Coverage	E-value	Identity	Accession Number
k99_618274_5	19A	472	aldehyde dehydrogenase [Candidatus Rokubacteria bacterium 13_1_40CM_69_27]	667	100%	0.0	68%	OLC14918.1
k99_231472_1	19B	461	putative aldehyde-dehydrogenase-like protein y4uC [Candidatus Methylomirabilis oxyfera]	682	100%	0.0	68%	CBE69421.1
k99_368232_2	19A	194	aldehyde dehydrogenase [Deltaproteobacteria bacterium GWA2_57_13]	260	100%	3.00E-82	65%	OGP19679.1
k99_155792_1	19A	470	putative aldehyde-dehydrogenase-like protein y4uC [Candidatus Methylomirabilis oxyfera]	664	100%	0.0	65%	CBE69421.1
k99_536255_2	19A	472	aldehyde dehydrogenase [Candidatus Rokubacteria bacterium 13_1_40CM_69_27]	662	100%	0.0	67%	OLC14918.1
k99_911916_1	19A	202	aldehyde dehydrogenase [Deltaproteobacteria bacterium GWA2_57_13]	275	100%	9.00E-88	64%	OGP19679.1
k99_962072_3	19A	472	putative aldehyde-dehydrogenase-like protein y4uC [Candidatus Methylomirabilis oxyfera]	666	99%	0.0	65%	CBE69421.1
k99_155792_1	19B	470	putative aldehyde-dehydrogenase-like protein y4uC [Candidatus Methylomirabilis oxyfera]	664	100%	0.0	65%	CBE69421.1
k99_536255_2	19B	472	aldehyde dehydrogenase [Candidatus Rokubacteria bacterium 13_1_40CM_69_27]	662	100%	0.0	67%	OLC14918.1
k99_856966_3	16C	180	putative oxidoreductase large subunit [uncultured Poribacteria bacterium 64K2]	357	100%	6.00E-117	97%	AAW84298.1
k99_924454_1	16C	582	putative oxidoreductase large subunit [uncultured Poribacteria bacterium 64K2]	1142	100%	0.0	95%	AAW84298.1
k99_924454_1	17	582	putative oxidoreductase large subunit [uncultured Poribacteria bacterium 64K2]	1142	100%	0.0	95%	AAW84298.1
k99_856966_3	19B	180	putative oxidoreductase large subunit [uncultured Poribacteria bacterium 64K2]	357	100%	6.00E-117	97%	AAW84298.1
k99_909490_1	25	642	putative oxidoreductase large subunit [uncultured Poribacteria bacterium 64K2]	1250	100%	0.0	94%	AAW84298.1
k99_910918_3	19B	292	putative oxidoreductase medium subunit [uncultured Poribacteria bacterium 64K2]	491	100%	3.00E-174	88%	AAW84296.1

Supplementary table S6. “Abundant” proteins (clusters > 5) in the sponge *Aplysina aerophoba*.

Cluster	No. proteins	BlastP hit	Query coverage	Identity	E-value
1	12	anti-anti-sigma factor [Acidobacteria bacterium RBG_16_70_10]	93%	35%	1.00E-13
2	8	hypothetical protein [Pseudohongiella spirulinae]	100%	56%	2.00E-132
3	6	tricarballylate dehydrogenase [Candidatus Entotheonella sp. TSY1]	97%	69%	0.0
4	6	aldehyde dehydrogenase [Candidatus Rokubacteria bacterium 13_1_40CM_69_27]	100%	68%	0.0
5	6	chaperonin GroL [Chloroflexi bacterium RBG_13_46_9]	99%	68%	9.00E-170
6	6	bleomycin hydrolase [Candidatus Synechococcus spongiarum 15L]	100%	100%	1.00E-131
7	6	bleomycin hydrolase [Candidatus Synechococcus spongiarum 15L]	100%	100%	3.00E-117
8	5	No Hit			
9	5	alcohol dehydrogenase [Candidatus Rokubacteria bacterium 13_2_20CM_70_12]	93%	59%	0.0
10	5	hypothetical protein ETSY1_00865 [Candidatus Entotheonella sp. TSY1]	100%	71%	0.0
11	5	hypothetical protein [Thalassobaculum salexigens]	99%	60%	0.0
12	5	60 kDa chaperonin [Chloroflexi bacterium OLB15]	96%	82%	0.0
13	5	peptide ABC transporter substrate-binding protein [Bradyrhizobium liaoningense]	99%	63%	0.0
14	5	possible trehalose/maltose binding protein [Roseobacter sp. SK209-2-6]	99%	45%	4.00E-140
15	5	hypothetical protein BV53_07080 [Candidatus Synechococcus spongiarum LMB bulk15N]	100%	99%	0.0
16	5	nitrate oxidoreductase subunit beta [Candidatus Nitrospira inopinata]	100%	95%	0.0
17	5	hypothetical protein ETSY1_23050 [Candidatus Entotheonella sp. TSY1]	100%	66%	0.0
18	5	quinoprotein ethanol dehydrogenase [Methyloversatilis universalis]	98%	49%	4.00E-111
19	5	chaperonin GroL [Candidatus Rokubacteria bacterium GWA2_70_23]	100%	71%	0.0
20	5	ABC transporter -binding protein [Rhodobacter sp. AKP1]	99%	53%	4.00E-140
21	5	cyclase [Betaproteobacteria bacterium SG8_39]	96%	59%	1.00E-140
22	5	hypothetical protein ETSY1_24210 [Candidatus Entotheonella sp. TSY1]	99%	69%	0.0
23	5	C4-dicarboxylate ABC transporter substrate-binding protein [Thalassobaculum salexigens]	97%	48%	2.00E-108
24	5	amino acid ABC transporter substrate-binding protein [Candidatus Entotheonella sp. TSY1]	96%	77%	0.0
25	5	hypothetical protein ETSY1_10675 [Candidatus Entotheonella sp. TSY1]	92%	75%	1.00E-173
26	5	ABC transporter permease [Rhodospirillales bacterium RIFCSPLOWO2_12_FULL_67_15]	97%	73%	1.00E-180
27	5	hypothetical protein ETSY2_04760 [Candidatus Entotheonella sp. TSY2]	97%	77%	0.0
28	5	hypothetical protein ETSY2_50085 [Candidatus Entotheonella sp. TSY2]	100%	59%	2.00E-131

29	5	TRAP-type mannitol/chloroaromatic compound transport system [Thalassobaculum litoreum DSM 18839]	98%	49%	2.00E-96
30	5	C4-dicarboxylate ABC transporter substrate-binding protein [Ruegeria pomeroyi]	95%	52%	3.00E-85
31	5	amino acid ABC transporter substrate-binding protein [Rhodobacteraceae bacterium HIMB11]	98%	69%	2.00E-117
32	5	fumarylacetoacetate hydrolase [Gemmatimonas sp. SG8_17]	98%	46%	5.00E-72
33	5	bleomycin hydrolase [Candidatus Synechococcus spongiarum 15L]	100%	100%	9.00E-116

Supplementary table S7. Annotation of carbohydrate active enzymes (CAZy) in the sponge proteome.

Protein_ID	Replicate	Family	E-value	Start_subject	End_subject	Start_query	End_query	Coverage_subject	Best Blastp Hit [taxa]	Score Blastp	Query Coverage	E-value	Identity	Accesion Number
k99_1172768_1	16C	AA3	1.2e-95	89	397	2	508	0.50	hypothetical protein ETSY2_18170 [Candidatus Entotheonella sp. TSY2]	756	99%	0.0	71%	ETX06269.1
k99_1187364_1	16C	AA3	1.5e-77	152	399	1	433	0.40	hypothetical protein ETSY1_25865 [Candidatus Entotheonella sp. TSY1]	660	99%	0.0	71%	ETW96626.1
k99_1254721_1	16C	AA3	8.1e-58	203	399	49	363	0.32	hypothetical protein ETSY2_18170 [Candidatus Entotheonella sp. TSY2]	533	99%	0.0	69%	ETX06269.1
k99_428039_2	16C	AA3	1.7e-90	89	400	2	516	0.50	hypothetical protein ETSY2_36430 [Candidatus Entotheonella sp. TSY2]	664	99%	0.0	60%	ETX01934.1
k99_500732_2	16C	AA3	1.8e-56	89	285	2	315	0.32	hypothetical protein ETSY1_09885 [Candidatus Entotheonella sp. TSY1]	412	97%	1.00E-138	59%	ETX00810.1
k99_581596_4	16C	AA3	1.7e-97	89	399	2	513	0.50	hypothetical protein ETSY2_36430 [Candidatus Entotheonella sp. TSY2]	774	99%	0.0	71%	ETX01934.1
k99_1187364_1	17	AA3	1.5e-77	152	399	1	433	0.40	hypothetical protein ETSY1_25865 [Candidatus Entotheonella sp. TSY1]	660	99%	0.0	71%	ETW96626.1
k99_1152699_3	19A	AA3	2.00E-49	92	402	13	522	0.50	2-keto-gluconate dehydrogenase [Bradyrhizobium sp. BR 10245]	759	100%	0.0	70%	WP_063697704.1
k99_1187364_1	19A	AA3	1.5e-77	152	399	1	433	0.40	hypothetical protein ETSY1_25865 [Candidatus Entotheonella sp. TSY1]	660	99%	0.0	71%	ETW96626.1
k99_1187364_1	19B	AA3	1.5e-77	152	399	1	433	0.40	hypothetical protein ETSY1_25865 [Candidatus Entotheonella sp. TSY1]	660	99%	0.0	71%	ETW96626.1
k99_380759_19	16C	AA7	8.3e-22	13	194	50	238	0.40	FAD-binding oxidoreductase [SAR116 cluster alpha proteobacterium HIMB100]	907	100%	0.0	89%	WP_009604064.1
k99_545796_1	16B	CBM32	4.5e-05	44	107	191	265	0.51	hypothetical protein [Candidatus Nitrosotenuis cloacae]	93.6	97%	1.00E-16	29%	WP_052755535.1
k99_997479_1	16B	CBM32	0.00045	50	109	721	790	0.48	hypothetical protein [Candidatus Nitrosotenuis cloacae]	99.8	94%	8.00E-18	29%	WP_052755486.1
k99_189657_2	16C	CBM32	4.2e-06	49	109	55	115	0.48	hypothetical protein [Candidatus Nitrosotenuis cloacae]	111	86%	3.00E-21	25%	WP_052755486.1
k99_997479_1	16C	CBM32	0.00045	50	109	721	790	0.48	hypothetical protein [Candidatus Nitrosotenuis cloacae]	99.8	94%	8.00E-18	29%	WP_052755486.1
k99_189657_2	17	CBM32	4.2e-06	49	109	55	115	0.48	hypothetical protein [Candidatus Nitrosotenuis cloacae]	111	86%	3.00E-21	25%	WP_052755486.1
k99_545796_1	17	CBM32	4.5e-05	44	107	191	265	0.51	hypothetical protein [Candidatus Nitrosotenuis cloacae]	93.6	97%	1.00E-16	29%	WP_052755535.1
k99_997479_1	17	CBM32	0.00045	50	109	721	790	0.48	hypothetical protein [Candidatus Nitrosotenuis cloacae]	99.8	94%	8.00E-18	29%	WP_052755486.1
k99_1136125_2	19A	CBM32	4.6e-06	49	109	388	449	0.48	hypothetical protein [Candidatus Nitrosotenuis cloacae]	128	76%	9.00E-27	27%	WP_052755486.1
k99_189657_2	19A	CBM32	4.2e-06	49	109	55	115	0.48	hypothetical protein [Candidatus Nitrosotenuis cloacae]	111	86%	3.00E-21	25%	WP_052755486.1
k99_189657_2	19B	CBM32	4.2e-06	49	109	55	115	0.48	hypothetical protein [Candidatus Nitrosotenuis cloacae]	111	86%	3.00E-21	25%	WP_052755486.1
k99_997479_1	25	CBM32	0.00045	50	109	721	790	0.48	hypothetical protein [Candidatus Nitrosotenuis cloacae]	99.8	94%	8.00E-18	29%	WP_052755486.1

k99_20117_1	17	CBM34	0.00012	30	79	258	307	0.41	leucine-rich repeat-containing protein 37B [Jaculus jaculus]	68.9	22%	4.00E-09	64%	XP_012805608.1
k99_1159524_1	16B	CBM37	2.9e-09	6	61	247	304	0.89	hypothetical protein [Gorillibacterium sp. SN4]	77.4	83%	7.00E-12	29%	WP_058302783.1
k99_1205730_2	16B	CBM37	0.00012	31	61	164	196	0.48	hypothetical protein [Candidatus Nitrosotenuis chungbukensis]	91.3	81%	3.00E-15	24%	WP_052347572.1
k99_1205730_2	16C	CBM37	0.00012	31	61	164	196	0.48	hypothetical protein [Candidatus Nitrosotenuis chungbukensis]	91.3	81%	3.00E-15	24%	WP_052347572.1
k99_626782_1	16C	CBM37	5.00E-05	3	54	142	197	0.82	hypothetical protein A3844_11225 [Paenibacillus sp. P26E]	145	86%	4.00E-33	32%	OKP87061.1
k99_683433_2	16C	CBM37	6.9e-05	8	61	1203	1259	0.85	hypothetical protein [Paenibacillus sp. ov031]	196	62%	2.00E-46	25%	WP_072733607.1
k99_683433_2	16C	CBM37	3.8e-05	9	61	1889	1940	0.84	hypothetical protein [Paenibacillus sp. ov031]	196	62%	2.00E-46	25%	WP_072733607.1
k99_1205730_2	17	CBM37	0.00012	31	61	164	196	0.48	hypothetical protein [Candidatus Nitrosotenuis chungbukensis]	91.3	81%	3.00E-15	24%	WP_052347572.1
k99_626782_1	17	CBM37	5.00E-05	3	54	142	197	0.82	hypothetical protein A3844_11225 [Paenibacillus sp. P26E]	145	86%	4.00E-33	32%	OKP87061.1
k99_751048_6	17	CBM37	0.00034	38	60	196	220	0.35	fibronectin type III domain-containing protein [Actinoplanes sp. N902-109]	80.1	98%	1.00E-11	29%	AGL16845.1
k99_189657_3	19A	CBM37	4.00E-10	6	60	84	140	0.87	collagen alpha-1(VII) chain [Elephantulus edwardii]	60.1	12%	1.00E-05	37%	XP_006893082.1
k99_626782_1	19A	CBM37	5.00E-05	3	54	142	197	0.82	hypothetical protein A3844_11225 [Paenibacillus sp. P26E]	145	86%	4.00E-33	32%	OKP87061.1
k99_932595_1	19A	CBM37	0.00017	39	61	51	73	0.35	hypothetical protein [Legionella santicrucis]	89.0	30%	4.00E-15	44%	WP_058512654.1
k99_189657_3	19B	CBM37	4.00E-10	6	60	84	140	0.87	collagen alpha-1(VII) chain [Elephantulus edwardii]	60.1	12%	1.00E-05	37%	XP_006893082.1
k99_626782_1	19B	CBM37	5.00E-05	3	54	142	197	0.82	hypothetical protein A3844_11225 [Paenibacillus sp. P26E]	145	86%	4.00E-33	32%	OKP87061.1
k99_932595_1	19B	CBM37	0.00017	39	61	51	73	0.35	hypothetical protein [Legionella santicrucis]	89.0	30%	4.00E-15	44%	WP_058512654.1
k99_1205730_2	25	CBM37	0.00012	31	61	164	196	0.48	hypothetical protein [Candidatus Nitrosotenuis chungbukensis]	91.3	81%	3.00E-15	24%	WP_052347572.1
k99_40787_1	17	CBM40	0.00012	90	175	162	236	0.47	hypothetical protein ACD_8C00080G0006 [uncultured bacterium]	62.0	88%	3.00E-07	26%	EKE19944.1
k99_262431_6	16C	CBM9	1.2e-07	1	174	38	260	0.95	sugar-binding protein [Alteromonas australica]	46.6	40%	0.023	32%	WP_044058497.1
k99_1221423_5	19A	CBM9	2.00E-05	32	92	86	149	0.33	hypothetical protein [Bacteroides ihuae]	57.4	93%	6.00E-06	24%	WP_071147673.1
k99_262431_6	19A	CBM9	1.2e-07	1	174	38	260	0.95	sugar-binding protein [Alteromonas australica]	46.6	40%	0.023	32%	WP_044058497.1
k99_436032_1	19A	CBM9	3.00E-18	21	167	96	282	0.80	hypothetical protein [Rhodopirellula baltica]	45.1	42%	0.021	29%	WP_037248028.1
k99_793166_3	19A	CBM9	4.5e-10	39	164	97	257	0.69	PKD domain-containing protein [Sphingomonas sp. PR090111-T3T-6A]	61.2	43%	5.00E-07	36%	WP_019832772.1
k99_903714_1	19A	CBM9	7.4e-06	1	78	41	130	0.42	PKD domain-containing protein [Candidatus Solibacter usitatus]	74.3	99%	5.00E-12	32%	WP_041857995.1
k99_1221423_5	19B	CBM9	2.00E-05	32	92	86	149	0.33	hypothetical protein [Bacteroides ihuae]	57.4	93%	6.00E-06	24%	WP_071147673.1
k99_262431_6	19B	CBM9	1.2e-07	1	174	38	260	0.95	sugar-binding protein [Alteromonas australica]	46.6	40%	0.023	32%	WP_044058497.1
k99_272231_2	16C	CE1	2.2e-06	7	211	24	226	0.90	hypothetical protein A3F90_00875 [Deltaproteobacteria bacterium RIFCSPLOWO2_12_FULL_60_19]	163	99%	3.00E-46	37%	OGQ79193.1
k99_272231_2	19B	CE1	2.2e-06	7	211	24	226	0.90	hypothetical protein A3F90_00875 [Deltaproteobacteria bacterium RIFCSPLOWO2_12_FULL_60_19]	163	99%	3.00E-46	37%	OGQ79193.1
k99_546451_1	17	CE10	7.2e-08	231	334	142	244	0.30	carboxymethylenebutenolidase [Candidatus Rokubacteria bacterium 13_1_40CM_4_69_5]	308	99%	4.00E-103	57%	OLC35454.1
k99_546451_1	19B	CE10	7.2e-08	231	334	142	244	0.30	carboxymethylenebutenolidase [Candidatus Rokubacteria bacterium 13_1_40CM_4_69_5]	308	99%	4.00E-103	57%	OLC35454.1

k99_534463_1	25	CE10	2.4e-08	231	334	63	165	0.30	carboxymethylenebutenolidase [Candidatus Rokubacteria bacterium 13_1_40CM_4_69_5]	203	98%	4.00E-63	57%	OLC35454.1
k99_546451_1	25	CE10	7.2e-08	231	334	142	244	0.30	carboxymethylenebutenolidase [Candidatus Rokubacteria bacterium 13_1_40CM_4_69_5]	308	99%	4.00E-103	57%	OLC35454.1
k99_745702_2	16C	CE3	3.3e-08	67	192	187	320	0.64	hydrolase GDSL [Paenibacillus senegalensis]	332	96%	8.00E-110	51%	WP_010277550.1
k99_893532_1	16C	CE3	9.7e-07	67	188	187	316	0.62	hydrolase GDSL [Paenibacillus senegalensis]	321	96%	7.00E-106	51%	WP_010277550.1
k99_1065762_3	16C	CE4	2.3e-19	24	127	44	149	0.79	polysaccharide deacetylase [Pseudomonas sp. ICMP 10191]	481	98%	4.00E-170	76%	WP_058416670.1
k99_1147702_2	16C	CE4	7.00E-20	26	127	47	149	0.78	polysaccharide deacetylase [Aneurinibacillus tyrosinisolvens]	513	98%	0.0	82%	WP_047152079.1
k99_1173408_1	16C	CE4	1.1e-18	24	127	44	149	0.79	polysaccharide deacetylase [Aneurinibacillus tyrosinisolvens]	429	98%	4.00E-150	78%	WP_047152079.1
k99_1182246_3	16C	CE4	1.00E-18	24	127	44	149	0.79	polysaccharide deacetylase [Pantoea sp. OXWO6B1]	485	98%	1.00E-171	79%	WP_063879658.1
k99_148695_1	16C	CE4	1.3e-19	24	127	34	139	0.79	polysaccharide deacetylase [Aneurinibacillus tyrosinisolvens]	486	100%	2.00E-172	80%	WP_047152079.1
k99_222753_1	16C	CE4	1.3e-19	24	127	34	139	0.79	polysaccharide deacetylase [Parageobacillus caldoxylosilyticus]	494	99%	2.00E-175	82%	WP_061579757.1
k99_782657_1	16C	CE4	3.5e-12	6	126	27	162	0.92	Chitin deacetylase 9-like protein [Daphnia magna]	286	96%	9.00E-91	41%	KZS21268.1
k99_900225_2	16C	CE4	9.3e-18	24	127	44	149	0.79	polysaccharide deacetylase [Pseudomonas syringae]	481	98%	3.00E-170	76%	WP_065835454.1
k99_782657_1	19A	CE4	3.5e-12	6	126	27	162	0.92	Chitin deacetylase 9-like protein [Daphnia magna]	286	96%	9.00E-91	41%	KZS21268.1
k99_1065762_3	19B	CE4	2.3e-19	24	127	44	149	0.79	polysaccharide deacetylase [Pseudomonas sp. ICMP 10191]	481	98%	4.00E-170	76%	WP_058416670.1
k99_1147702_2	19B	CE4	7.00E-20	26	127	47	149	0.78	polysaccharide deacetylase [Aneurinibacillus tyrosinisolvens]	513	98%	0.0	82%	WP_047152079.1
k99_1173408_1	19B	CE4	1.1e-18	24	127	44	149	0.79	hypothetical protein [Aneurinibacillus terranovensis]	431	100%	4.00E-151	78%	WP_027415559.1
k99_1182246_3	19B	CE4	1.00E-18	24	127	44	149	0.79	polysaccharide deacetylase [Pantoea sp. OXWO6B1]	485	98%	1.00E-171	79%	WP_063879658.1
k99_148695_1	19B	CE4	1.3e-19	24	127	34	139	0.79	polysaccharide deacetylase [Aneurinibacillus tyrosinisolvens]	486	100%	2.00E-172	80%	WP_047152079.1
k99_222753_1	19B	CE4	1.3e-19	24	127	34	139	0.79	polysaccharide deacetylase [Parageobacillus caldoxylosilyticus]	494	99%	2.00E-175	82%	WP_061579757.1
k99_782657_1	19B	CE4	3.5e-12	6	126	27	162	0.92	Chitin deacetylase 9-like protein [Daphnia magna]	286	96%	9.00E-91	41%	KZS21268.1
k99_900225_2	19B	CE4	9.3e-18	24	127	44	149	0.79	polysaccharide deacetylase [Pantoea stewartii]	483	98%	8.00E-171	78%	WP_033741691.1
k99_1069468_1	19A	cohesin	5.00E-09	13	124	1	104	0.83	Secreted protein [Candidatus Daviesbacteria bacterium GW2011_GWB1_36_5]	89.0	44%	6.00E-17	48%	KKQ09860.1
k99_1247336_4	16C	GH109	8.6e-11	1	118	3	115	0.93	hypothetical protein AUJ96_12415 [Armatimonadetes bacterium CG2_30_66_41]	575	100%	0.0	74%	OIP04581.1
k99_558204_3	16C	GH109	4.3e-12	1	118	3	115	0.93	hypothetical protein AUJ96_12415 [Armatimonadetes bacterium CG2_30_66_41]	577	99%	0.0	75%	OIP04581.1
k99_614873_1	16C	GH109	1.8e-05	71	118	24	75	0.37	hypothetical protein AUJ96_12415 [Armatimonadetes bacterium CG2_30_66_41]	511	100%	5.00E-180	75%	OIP04581.1
k99_840834_3	16C	GH109	8.7e-11	1	118	3	115	0.93	hypothetical protein AUJ96_12415 [Armatimonadetes bacterium CG2_30_66_41]	558	100%	0.0	75%	OIP04581.1
k99_929070_4	16C	GH109	9.00E-11	1	118	3	115	0.93	hypothetical protein AUJ96_12415 [Armatimonadetes bacterium CG2_30_66_41]	574	100%	0.0	74%	OIP04581.1
k99_940348_3	16C	GH109	1.5e-15	1	122	5	129	0.96	hypothetical protein AUJ96_08325 [Armatimonadetes bacterium CG2_30_66_41]	329	100%	4.00E-103	44%	OIP06923.1
k99_973966_3	16C	GH109	1.1e-10	1	118	3	115	0.93	hypothetical protein AUJ96_12415 [Armatimonadetes bacterium CG2_30_66_41]	578	99%	0.0	75%	OIP04581.1
k99_812122_6	19A	GH109	1.1e-06	1	47	5	52	0.37	hypothetical protein [Paenibacillus daejeonensis]	164	95%	2.00E-43	31%	WP_020617322.1

k99_812122_6	19A	GH109	1.9e-09	36	118	75	153	0.65	hypothetical protein [Paenibacillus daejeonensis]	164	95%	2.00E-43	31%	WP_020617322.1
k99_985772_1	19A	GH109	3.7e-07	1	48	5	53	0.37	dehydrogenase [Lentisphaerae bacterium GWF2_50_93]	154	99%	4.00E-41	35%	OGV55436.1
k99_985772_1	19A	GH109	1.1e-09	29	118	61	153	0.71	dehydrogenase [Lentisphaerae bacterium GWF2_50_93]	154	99%	4.00E-41	35%	OGV55436.1
k99_103996_3	19B	GH109	2.9e-15	1	122	5	129	0.96	hypothetical protein AUJ92_21775 [Armatimonadetes bacterium CG2_30_59_28]	335	98%	1.00E-109	46%	OIO89361.1
k99_1152758_2	19B	GH109	3.9e-14	1	119	5	126	0.94	hypothetical protein AUJ92_21775 [Armatimonadetes bacterium CG2_30_59_28]	336	98%	5.00E-110	46%	OIO89361.1
k99_1222047_2	19B	GH109	3.1e-16	1	123	6	131	0.97	hypothetical protein AUJ92_21775 [Armatimonadetes bacterium CG2_30_59_28]	335	98%	9.00E-110	45%	OIO89361.1
k99_1247336_4	19B	GH109	8.6e-11	1	118	3	115	0.93	hypothetical protein AUJ96_12415 [Armatimonadetes bacterium CG2_30_66_41]	575	100%	0.0	74%	OIP04581.1
k99_175454_1	19B	GH109	8.4e-14	1	110	4	104	0.87	hypothetical protein A3F84_03835 [Candidatus Handelsmanbacteria bacterium RIFCSPLOWO2_12_FULL_64_10]	301	99%	4.00E-98	48%	OGG52202.1
k99_415859_1	19B	GH109	1.6e-13	1	109	4	103	0.86	hypothetical protein A3F84_03835 [Candidatus Handelsmanbacteria bacterium RIFCSPLOWO2_12_FULL_64_10]	301	99%	2.00E-98	48%	OGG52202.1
k99_558204_3	19B	GH109	4.3e-12	1	118	3	115	0.93	hypothetical protein AUJ96_12415 [Armatimonadetes bacterium CG2_30_66_41]	577	99%	0.0	75%	OIP04581.1
k99_614873_1	19B	GH109	1.8e-05	71	118	24	75	0.37	hypothetical protein AUJ96_12415 [Armatimonadetes bacterium CG2_30_66_41]	511	100%	5.00E-180	75%	OIP04581.1
k99_840834_3	19B	GH109	8.7e-11	1	118	3	115	0.93	hypothetical protein AUJ96_12415 [Armatimonadetes bacterium CG2_30_66_41]	558	100%	0.0	75%	OIP04581.1
k99_929070_4	19B	GH109	9.00E-11	1	118	3	115	0.93	hypothetical protein AUJ96_12415 [Armatimonadetes bacterium CG2_30_66_41]	574	100%	0.0	74%	OIP04581.1
k99_973966_3	19B	GH109	1.1e-10	1	118	3	115	0.93	hypothetical protein AUJ96_12415 [Armatimonadetes bacterium CG2_30_66_41]	578	99%	0.0	75%	OIP04581.1
k99_614319_2	16B	GH116	8.4e-15	66	212	404	550	0.40	hypothetical protein AMS20_01875 [Gemmatimonas sp. SG8_28]	929	87%	0.0	59%	KPK08562.1
k99_759419_1	16B	GH3	2.2e-58	6	216	99	324	0.97	beta-N-acetylglucosaminidase [Formosa sp. Hel3_A1_48]	1479	100%	0.0	72%	WP_069675134.1
k99_753534_1	16C	GH38	4.1e-37	102	269	2	209	0.62	Lysosomal alpha-mannosidase [Exaiptasia pallida]	345	100%	5.00E-110	59%	KXJ23287.1
k99_753534_1	17	GH38	4.1e-37	102	269	2	209	0.62	Lysosomal alpha-mannosidase [Exaiptasia pallida]	345	100%	5.00E-110	59%	KXJ23287.1
k99_753534_1	19B	GH38	4.1e-37	102	269	2	209	0.62	Lysosomal alpha-mannosidase [Exaiptasia pallida]	345	100%	5.00E-110	59%	KXJ23287.1
k99_1222687_1	19B	GH74	1.1e-12	43	115	10	78	0.31	hypothetical protein ETSY2_48545 [Candidatus Entotheonella sp. TSY2]	455	99%	4.00E-158	65%	ETW95178.1
k99_1222687_1	19B	GH74	2.1e-10	41	111	147	216	0.30	hypothetical protein ETSY2_48545 [Candidatus Entotheonella sp. TSY2]	455	0.99	4E-158	0.65	ETW95178.1
k99_824113_6	19B	GH74	1.1e-12	43	114	10	77	0.30	hypothetical protein ETSY2_48545 [Candidatus Entotheonella sp. TSY2]	450	99%	5.00E-156	64%	ETW95178.1
k99_824113_6	19B	GH74	1.3e-10	38	111	143	216	0.31	hypothetical protein ETSY2_48545 [Candidatus Entotheonella sp. TSY2]	450	0.99	5E-156	0.64	ETW95178.1
k99_824113_6	19B	GH74	1.4e-09	43	113	248	315	0.30	hypothetical protein ETSY2_48545 [Candidatus Entotheonella sp. TSY2]	450	0.99	5E-156	0.64	ETW95178.1
k99_689967_2	19A	GH95	2.5e-303	3	714	5	735	0.98	alpha-L-fucosidase [Rhodopirellula maiorica]	867	94%	0.0	59%	WP_008698035.1
k99_257867_2	16C	GT26	6.00E-06	40	108	166	236	0.40	branched-chain amino acid ABC transporter substrate-binding protein [Candidatus Tectomicrobia bacterium RIFCSPLOWO2_02_FULL_70_19]	579	94%	0.0	70%	OGL64366.1
k99_1038292_1	19A	GT26	4.7e-06	41	111	25	96	0.41	branched-chain amino acid ABC transporter substrate-binding protein [Candidatus Tectomicrobia bacterium RIFCSPLOWO2_02_FULL_70_19]	397	100%	2.00E-135	66%	OGL64366.1
k99_850093_2	19A	SLH	8.7e-06	1	41	87	128	0.95	adhesin [Paenibacillus kribbensis]	72.8	21%	1.00E-09	37%	WP_068498700.1
k99_850093_2	19A	SLH	2.8e-05	1	41	151	190	0.95	adhesin [Paenibacillus kribbensis]	72.8	21%	1.00E-09	37%	WP_068498700.1

Supplementary table S8. Sequencing effort of this study and overview of reads mapped to metagenome-assembled contigs ≥ 1000 bp.

Sample Habitat	Individual	Replicate	Interleaved fasta file size (GB)	Metagenome No. Reads after QC	No. Mapped Metagenomic Reads onto assembly	% mapped Metagenomic reads onto assembly
A. aerophoba	16	A	6	44816170	20216754	45.11
A. aerophoba	21	A	5.2	38335413	15193908	39.63
A. aerophoba	22	A	5.3	39606255	13946183	35.21
A. aerophoba	22	B	6	44448937	17483131	39.33
A. aerophoba	22	C	5.2	38351903	19671751	51.29
A. aerophoba	23	A	5.3	39359086	17499080	44.46
Sea water	5	A	5.2	22622178	9198510	40.46
Sea water	7	A	5.1	21881173	10491414	46.66
Sea water	9	A	5.5	23814456	8864676	35.61
Gene marker approach-based taxonomic overview of assembly using Phylosift						
Sponge	Number of contigs	% contigs assigned to Bacteria	% contigs assigned to Archaea	% contigs assigned to Eukaryota	% Unassigned contigs	
A. aerophoba	273718	91	3	5.3	0.7	
